# Genetic Modulation of Lipid Profiles following Lifestyle Modification or Metformin Treatment: The Diabetes Prevention Program

**DOI:** 10.1371/journal.pgen.1002895

**Published:** 2012-08-30

**Authors:** Toni I. Pollin, Tamara Isakova, Kathleen A. Jablonski, Paul I. W. de Bakker, Andrew Taylor, Jarred McAteer, Qing Pan, Edward S. Horton, Linda M. Delahanty, David Altshuler, Alan R. Shuldiner, Ronald B. Goldberg, Jose C. Florez, Paul W. Franks, Diabetes Prevention Program Research Group, George A. Bray, Iris W. Culbert, Catherine M. Champagne, Barbara Eberhardt, Frank Greenway, Fonda G. Guillory, April A. Herbert, Michael L. Jeffirs, Betty M. Kennedy, Jennifer C. Lovejoy, Laura H. Morris, Lee E. Melancon, Donna Ryan, Deborah A. Sanford, Kenneth G. Smith, Lisa L. Smith, Julia A. St. Amant, Richard T. Tulley, Paula C. Vicknair, Donald Williamson, Jeffery J. Zachwieja, Kenneth S. Polonsky, Janet Tobian, David Ehrmann, Margaret J. Matulik, Bart Clark, Kirsten Czech, Catherine DeSandre, Ruthanne Hilbrich, Wylie McNabb, Ann R. Semenske, Jose F. Caro, Pamela G. Watson, Barry J. Goldstein, Kellie A. Smith, Jewel Mendoza, Renee Liberoni, Constance Pepe, John Spandorfer, Richard P. Donahue, Ronald B. Goldberg, Ronald Prineas, Patricia Rowe, Jeanette Calles, Paul Cassanova-Romero, Hermes J. Florez, Anna Giannella, Lascelles Kirby, Carmen Larreal, Valerie McLymont, Jadell Mendez, Juliet Ojito, Arlette Perry, Patrice Saab, Steven M. Haffner, Maria G. Montez, Carlos Lorenzo, Arlene Martinez, Richard F. Hamman, Patricia V. Nash, Lisa Testaverde, Denise R. Anderson, Larry B. Ballonoff, Alexis Bouffard, B. Ned Calonge, Lynne Delve, Martha Farago, James O. Hill, Shelley R. Hoyer, Bonnie T. Jortberg, Dione Lenz, Marsha Miller, David W. Price, Judith G. Regensteiner, Helen Seagle, Carissa M. Smith, Sheila C. Steinke, Brent VanDorsten, Edward S. Horton, Kathleen E. Lawton, Ronald A. Arky, Marybeth Bryant, Jacqueline P. Burke, Enrique Caballero, Karen M. Callaphan, Om P. Ganda, Therese Franklin, Sharon D. Jackson, Alan M. Jacobsen, Alan M. Jacobsen, Lyn M. Kula, Margaret Kocal, Maureen A. Malloy, Maryanne Nicosia, Cathryn F. Oldmixon, Jocelyn Pan, Marizel Quitingon, Stacy Rubtchinsky, Ellen W. Seely, Dana Schweizer, Donald Simonson, Fannie Smith, Caren G. Solomon, James Warram, Steven E. Kahn, Brenda K. Montgomery, Wilfred Fujimoto, Robert H. Knopp, Edward W. Lipkin, Michelle Marr, Dace Trence, Abbas E. Kitabchi, Mary E. Murphy, William B. Applegate, Michael Bryer-Ash, Sandra L. Frieson, Raed Imseis, Helen Lambeth, Lynne C. Lichtermann, Hooman Oktaei, Lily M.K. Rutledge, Amy R. Sherman, Clara M. Smith, Judith E. Soberman, Beverly Williams-Cleaves, Boyd E. Metzger, Mariana K. Johnson, Catherine Behrends, Michelle Cook, Marian Fitzgibbon, Mimi M. Giles, Deloris Heard, Cheryl K.H. Johnson, Diane Larsen, Anne Lowe, Megan Lyman, David McPherson, Mark E. Molitch, Thomas Pitts, Renee Reinhart, Susan Roston, Pamela A. Schinleber, David M. Nathan, Charles McKitrick, Heather Turgeon, Kathy Abbott, Ellen Anderson, Laurie Bissett, Enrico Cagliero, Jose C. Florez, Linda Delahanty, Valerie Goldman, Alexandra Poulos, Jerrold M. Olefsky, Mary Lou Carrion-Petersen, Elizabeth Barrett-Connor, Steven V. Edelman, Robert R. Henry, Javiva Horne, Simona Szerdi Janesch, Diana Leos, Sundar Mudaliar, William Polonsky, Jean Smith, Karen Vejvoda, F. Xavier Pi-Sunyer, Jane E. Lee, David B. Allison, Nancy J. Aronoff, Jill P. Crandall, Sandra T. Foo, Carmen Pal, Kathy Parkes, Mary Beth Pena, Ellen S. Rooney, Gretchen E.H. Van Wye, Kristine A. Viscovich, David G. Marrero, Melvin J. Prince, Susie M. Kelly, Yolanda F. Dotson, Edwin S. Fineberg, John C Guare, Angela M. Hadden, James M. Ignaut, Marcia L. Jackson, Marion S. Kirkman, Kieren J. Mather, Beverly D. Porter, Paris J. Roach, Nancy D. Rowland, Madelyn L. Wheeler, Robert E. Ratner, Gretchen Youssef, Sue Shapiro, Catherine Bavido-Arrage, Geraldine Boggs, Marjorie Bronsord, Ernestine Brown, Wayman W. Cheatham, Susan Cola, Cindy Evans, Peggy Gibbs, Tracy Kellum, Claresa Levatan, Asha K. Nair, Maureen Passaro, Gabriel Uwaifo, Mohammed F. Saad, Maria Budget, Sujata Jinagouda, Khan Akbar, Claudia Conzues, Perpetua Magpuri, Kathy Ngo, Amer Rassam, Debra Waters, Kathy Xapthalamous, Julio V. Santiago, Samuel Dagogo-Jack, Neil H. White, Samia Das, Ana Santiago, Angela Brown, Edwin Fisher, Emma Hurt, Tracy Jones, Michelle Kerr, Lucy Ryder, Cormarie Wernimont, Christopher D. Saudek, Vanessa Bradley, Emily Sullivan, Tracy Whittington, Caroline Abbas, Frederick L. Brancati, Jeanne M. Clark, Jeanne B. Charleston, Janice Freel, Katherine Horak, Dawn Jiggetts, Deloris Johnson, Hope Joseph, Kimberly Loman, Henry Mosley, Richard R. Rubin, Alafia Samuels, Kerry J. Stewart, Paula Williamson, David S. Schade, Karwyn S. Adams, Carolyn Johannes, Leslie F. Atler, Patrick J. Boyle, Mark R. Burge, Janene L. Canady, Lisa Chai, Ysela Gonzales, Doris A. Hernandez-McGinnis, Patricia Katz, Carolyn King, Amer Rassam, Sofya Rubinchik, Willette Senter, Debra Waters, Harry Shamoon, Janet O. Brown, Elsie Adorno, Liane Cox, Jill Crandall, Helena Duffy, Samuel Engel, Allison Friedler, Crystal J. Howard-Century, Stacey Kloiber, Nadege Longchamp, Helen Martinez, Dorothy Pompi, Jonathan Scheindlin, Elissa Violino, Elizabeth Walker, Judith Wylie-Rosett, Elise Zimmerman, Joel Zonszein, Trevor Orchard, Rena R. Wing, Gaye Koenning, M. Kaye Kramer, Susan Barr, Miriam Boraz, Lisa Clifford, Rebecca Culyba, Marlene Frazier, Ryan Gilligan, Susan Harrier, Louann Harris, Susan Jeffries, Andrea Kriska, Qurashia Manjoo, Monica Mullen, Alicia Noel, Amy Otto, Linda Semler, Cheryl F. Smith, Marie Smith, Elizabeth Venditti, Valarie Weinzierl, Katherine V. Williams, Tara Wilson, Richard F. Arakaki, Renee W. Latimer, Narleen K. Baker-Ladao, Ralph Beddow, Lorna Dias, Jillian Inouye, Marjorie K. Mau, Kathy Mikami, Pharis Mohideen, Sharon K. Odom, Raynette U. Perry, William C. Knowler, Norman Cooeyate, Mary A. Hoskin, Carol A. Percy, Kelly J. Acton, Vickie L. Andre, Rosalyn Barber, Shandiin Begay, Peter H. Bennett, Mary Beth Benson, Evelyn C. Bird, Brenda A. Broussard, Marcella Chavez, Tara Dacawyma, Matthew S. Doughty, Roberta Duncan, Cyndy Edgerton, Jacqueline M. Ghahate, Justin Glass, Martia Glass, Dorothy Gohdes, Wendy Grant, Robert L. Hanson, Ellie Horse, Louise E. Ingraham, Merry Jackson, Priscilla Jay, Roylen S. Kaskalla, David Kessler, Kathleen M. Kobus, Jonathan Krakoff, Catherine Manus, Sara Michaels, Tina Morgan, Yolanda Nashboo, Julie A. Nelson, Steven Poirier, Evette Polczynski, Mike Reidy, Jeanine Roumain, Debra Rowse, Sandra Sangster, Janet Sewenemewa, Darryl Tonemah, Charlton Wilson, Michelle Yazzie, Raymond Bain, Sarah Fowler, Tina Brenneman, Solome Abebe, Julie Bamdad, Jackie Callaghan, Sharon L. Edelstein, Yuping Gao, Kristina L. Grimes, Nisha Grover, Lori Haffner, Steve Jones, Tara L. Jones, Richard Katz, John M. Lachin, Pamela Mucik, Robert Orlosky, James Rochon, Alla Sapozhnikova, Hanna Sherif, Charlotte Stimpson, Marinella Temprosa, Fredricka Walker-Murray, Santica Marcovina, Greg Strylewicz, F. Alan Aldrich, Dan O'Leary, Elizabeth Stamm, Pentti Rautaharju, Ronald J. Prineas, Teresa Alexander, Charles Campbell, Sharon Hall, Yabing Li, Margaret Mills, Nancy Pemberton, Farida Rautaharju, Zhuming Zhang, Elizabeth Mayer-Davis, Robert R. Moran, Ted Ganiats, Kristin David, Andrew J. Sarkin, R. Eastman, Judith Fradkin, Sanford Garfield, Edward Gregg, Ping Zhang, William Herman, Jose C. Florez, David Altshuler, Paul I.W. de Bakker, Paul W. Franks, Robert L. Hanson, Kathleen Jablonski, William C. Knowler, Jarred B. McAteer, Toni I. Pollin, Alan R. Shuldiner

**Affiliations:** Pennington Biomedical Research Center (Baton Rouge, LA); Pennington Biomedical Research Center (Baton Rouge, LA); Pennington Biomedical Research Center (Baton Rouge, LA); Pennington Biomedical Research Center (Baton Rouge, LA); Pennington Biomedical Research Center (Baton Rouge, LA); Pennington Biomedical Research Center (Baton Rouge, LA); Pennington Biomedical Research Center (Baton Rouge, LA); Pennington Biomedical Research Center (Baton Rouge, LA); Pennington Biomedical Research Center (Baton Rouge, LA); Pennington Biomedical Research Center (Baton Rouge, LA); Pennington Biomedical Research Center (Baton Rouge, LA); Pennington Biomedical Research Center (Baton Rouge, LA); Pennington Biomedical Research Center (Baton Rouge, LA); Pennington Biomedical Research Center (Baton Rouge, LA); Pennington Biomedical Research Center (Baton Rouge, LA); Pennington Biomedical Research Center (Baton Rouge, LA); Pennington Biomedical Research Center (Baton Rouge, LA); Pennington Biomedical Research Center (Baton Rouge, LA); Pennington Biomedical Research Center (Baton Rouge, LA); Pennington Biomedical Research Center (Baton Rouge, LA); Pennington Biomedical Research Center (Baton Rouge, LA); University of Chicago (Chicago, IL); University of Chicago (Chicago, IL); University of Chicago (Chicago, IL); University of Chicago (Chicago, IL); University of Chicago (Chicago, IL); University of Chicago (Chicago, IL); University of Chicago (Chicago, IL); University of Chicago (Chicago, IL); University of Chicago (Chicago, IL); University of Chicago (Chicago, IL); Jefferson Medical College (Philadelphia, PA); Jefferson Medical College (Philadelphia, PA); Jefferson Medical College (Philadelphia, PA); Jefferson Medical College (Philadelphia, PA); Jefferson Medical College (Philadelphia, PA); Jefferson Medical College (Philadelphia, PA); Jefferson Medical College (Philadelphia, PA); Jefferson Medical College (Philadelphia, PA); University of Miami (Miami, FL); University of Miami (Miami, FL); University of Miami (Miami, FL); University of Miami (Miami, FL); University of Miami (Miami, FL); University of Miami (Miami, FL); University of Miami (Miami, FL); University of Miami (Miami, FL); University of Miami (Miami, FL); University of Miami (Miami, FL); University of Miami (Miami, FL); University of Miami (Miami, FL); University of Miami (Miami, FL); University of Miami (Miami, FL); University of Miami (Miami, FL); The University of Texas Health Science Center (San Antonio, TX); The University of Texas Health Science Center (San Antonio, TX); The University of Texas Health Science Center (San Antonio, TX); The University of Texas Health Science Center (San Antonio, TX); University of Colorado (Denver, CO); University of Colorado (Denver, CO); University of Colorado (Denver, CO); University of Colorado (Denver, CO); University of Colorado (Denver, CO); University of Colorado (Denver, CO); University of Colorado (Denver, CO); University of Colorado (Denver, CO); University of Colorado (Denver, CO); University of Colorado (Denver, CO); University of Colorado (Denver, CO); University of Colorado (Denver, CO); University of Colorado (Denver, CO); University of Colorado (Denver, CO); University of Colorado (Denver, CO); University of Colorado (Denver, CO); University of Colorado (Denver, CO); University of Colorado (Denver, CO); University of Colorado (Denver, CO); University of Colorado (Denver, CO); Joslin Diabetes Center (Boston, MA); Joslin Diabetes Center (Boston, MA); Joslin Diabetes Center (Boston, MA); Joslin Diabetes Center (Boston, MA); Joslin Diabetes Center (Boston, MA); Joslin Diabetes Center (Boston, MA); Joslin Diabetes Center (Boston, MA); Joslin Diabetes Center (Boston, MA); Joslin Diabetes Center (Boston, MA); Joslin Diabetes Center (Boston, MA); Joslin Diabetes Center (Boston, MA); Joslin Diabetes Center (Boston, MA); Joslin Diabetes Center (Boston, MA); Joslin Diabetes Center (Boston, MA); Joslin Diabetes Center (Boston, MA); Joslin Diabetes Center (Boston, MA); Joslin Diabetes Center (Boston, MA); Joslin Diabetes Center (Boston, MA); Joslin Diabetes Center (Boston, MA); Joslin Diabetes Center (Boston, MA); Joslin Diabetes Center (Boston, MA); Joslin Diabetes Center (Boston, MA); Joslin Diabetes Center (Boston, MA); Joslin Diabetes Center (Boston, MA); Joslin Diabetes Center (Boston, MA); Joslin Diabetes Center (Boston, MA); VA Puget Sound Health Care System and University of Washington (Seattle, WA); VA Puget Sound Health Care System and University of Washington (Seattle, WA); VA Puget Sound Health Care System and University of Washington (Seattle, WA); VA Puget Sound Health Care System and University of Washington (Seattle, WA); VA Puget Sound Health Care System and University of Washington (Seattle, WA); VA Puget Sound Health Care System and University of Washington (Seattle, WA); VA Puget Sound Health Care System and University of Washington (Seattle, WA); University of Tennessee (Memphis, TN); University of Tennessee (Memphis, TN); University of Tennessee (Memphis, TN); University of Tennessee (Memphis, TN); University of Tennessee (Memphis, TN); University of Tennessee (Memphis, TN); University of Tennessee (Memphis, TN); University of Tennessee (Memphis, TN); University of Tennessee (Memphis, TN); University of Tennessee (Memphis, TN); University of Tennessee (Memphis, TN); University of Tennessee (Memphis, TN); University of Tennessee (Memphis, TN); University of Tennessee (Memphis, TN); Northwestern University's Feinberg School of Medicine (Chicago, IL); Northwestern University's Feinberg School of Medicine (Chicago, IL); Northwestern University's Feinberg School of Medicine (Chicago, IL); Northwestern University's Feinberg School of Medicine (Chicago, IL); Northwestern University's Feinberg School of Medicine (Chicago, IL); Northwestern University's Feinberg School of Medicine (Chicago, IL); Northwestern University's Feinberg School of Medicine (Chicago, IL); Northwestern University's Feinberg School of Medicine (Chicago, IL); Northwestern University's Feinberg School of Medicine (Chicago, IL); Northwestern University's Feinberg School of Medicine (Chicago, IL); Northwestern University's Feinberg School of Medicine (Chicago, IL); Northwestern University's Feinberg School of Medicine (Chicago, IL); Northwestern University's Feinberg School of Medicine (Chicago, IL); Northwestern University's Feinberg School of Medicine (Chicago, IL); Northwestern University's Feinberg School of Medicine (Chicago, IL); Northwestern University's Feinberg School of Medicine (Chicago, IL); Northwestern University's Feinberg School of Medicine (Chicago, IL); Massachusetts General Hospital (Boston, MA); Massachusetts General Hospital (Boston, MA); Massachusetts General Hospital (Boston, MA); Massachusetts General Hospital (Boston, MA); Massachusetts General Hospital (Boston, MA); Massachusetts General Hospital (Boston, MA); Massachusetts General Hospital (Boston, MA); Massachusetts General Hospital (Boston, MA); Massachusetts General Hospital (Boston, MA); Massachusetts General Hospital (Boston, MA); Massachusetts General Hospital (Boston, MA); University of California-San Diego (San Diego, CA); University of California-San Diego (San Diego, CA); University of California-San Diego (San Diego, CA); University of California-San Diego (San Diego, CA); University of California-San Diego (San Diego, CA); University of California-San Diego (San Diego, CA); University of California-San Diego (San Diego, CA); University of California-San Diego (San Diego, CA); University of California-San Diego (San Diego, CA); University of California-San Diego (San Diego, CA); University of California-San Diego (San Diego, CA); University of California-San Diego (San Diego, CA); St. Luke's-Roosevelt Hospital (New York, NY); St. Luke's-Roosevelt Hospital (New York, NY); St. Luke's-Roosevelt Hospital (New York, NY); St. Luke's-Roosevelt Hospital (New York, NY); St. Luke's-Roosevelt Hospital (New York, NY); St. Luke's-Roosevelt Hospital (New York, NY); St. Luke's-Roosevelt Hospital (New York, NY); St. Luke's-Roosevelt Hospital (New York, NY); St. Luke's-Roosevelt Hospital (New York, NY); St. Luke's-Roosevelt Hospital (New York, NY); St. Luke's-Roosevelt Hospital (New York, NY); St. Luke's-Roosevelt Hospital (New York, NY); Indiana University (Indianapolis, IN); Indiana University (Indianapolis, IN); Indiana University (Indianapolis, IN); Indiana University (Indianapolis, IN); Indiana University (Indianapolis, IN); Indiana University (Indianapolis, IN); Indiana University (Indianapolis, IN); Indiana University (Indianapolis, IN); Indiana University (Indianapolis, IN); Indiana University (Indianapolis, IN); Indiana University (Indianapolis, IN); Indiana University (Indianapolis, IN); Indiana University (Indianapolis, IN); Indiana University (Indianapolis, IN); Indiana University (Indianapolis, IN); Medstar Research Institute (Washington, DC); Medstar Research Institute (Washington, DC); Medstar Research Institute (Washington, DC); Medstar Research Institute (Washington, DC); Medstar Research Institute (Washington, DC); Medstar Research Institute (Washington, DC); Medstar Research Institute (Washington, DC); Medstar Research Institute (Washington, DC); Medstar Research Institute (Washington, DC); Medstar Research Institute (Washington, DC); Medstar Research Institute (Washington, DC); Medstar Research Institute (Washington, DC); Medstar Research Institute (Washington, DC); Medstar Research Institute (Washington, DC); Medstar Research Institute (Washington, DC); Medstar Research Institute (Washington, DC); University of Southern California/UCLA Research Center (Alhambra, CA); University of Southern California/UCLA Research Center (Alhambra, CA); University of Southern California/UCLA Research Center (Alhambra, CA); University of Southern California/UCLA Research Center (Alhambra, CA); University of Southern California/UCLA Research Center (Alhambra, CA); University of Southern California/UCLA Research Center (Alhambra, CA); University of Southern California/UCLA Research Center (Alhambra, CA); University of Southern California/UCLA Research Center (Alhambra, CA); University of Southern California/UCLA Research Center (Alhambra, CA); University of Southern California/UCLA Research Center (Alhambra, CA); Washington University (St. Louis, MO); Washington University (St. Louis, MO); Washington University (St. Louis, MO); Washington University (St. Louis, MO); Washington University (St. Louis, MO); Washington University (St. Louis, MO); Washington University (St. Louis, MO); Washington University (St. Louis, MO); Washington University (St. Louis, MO); Washington University (St. Louis, MO); Washington University (St. Louis, MO); Johns Hopkins School of Medicine (Baltimore, MD); Johns Hopkins School of Medicine (Baltimore, MD); Johns Hopkins School of Medicine (Baltimore, MD); Johns Hopkins School of Medicine (Baltimore, MD); Johns Hopkins School of Medicine (Baltimore, MD); Johns Hopkins School of Medicine (Baltimore, MD); Johns Hopkins School of Medicine (Baltimore, MD); Johns Hopkins School of Medicine (Baltimore, MD); Johns Hopkins School of Medicine (Baltimore, MD); Johns Hopkins School of Medicine (Baltimore, MD); Johns Hopkins School of Medicine (Baltimore, MD); Johns Hopkins School of Medicine (Baltimore, MD); Johns Hopkins School of Medicine (Baltimore, MD); Johns Hopkins School of Medicine (Baltimore, MD); Johns Hopkins School of Medicine (Baltimore, MD); Johns Hopkins School of Medicine (Baltimore, MD); Johns Hopkins School of Medicine (Baltimore, MD); Johns Hopkins School of Medicine (Baltimore, MD); Johns Hopkins School of Medicine (Baltimore, MD); University of New Mexico (Albuquerque, NM); University of New Mexico (Albuquerque, NM); University of New Mexico (Albuquerque, NM); University of New Mexico (Albuquerque, NM); University of New Mexico (Albuquerque, NM); University of New Mexico (Albuquerque, NM); University of New Mexico (Albuquerque, NM); University of New Mexico (Albuquerque, NM); University of New Mexico (Albuquerque, NM); University of New Mexico (Albuquerque, NM); University of New Mexico (Albuquerque, NM); University of New Mexico (Albuquerque, NM); University of New Mexico (Albuquerque, NM); University of New Mexico (Albuquerque, NM); University of New Mexico (Albuquerque, NM); University of New Mexico (Albuquerque, NM); Albert Einstein College of Medicine (Bronx, NY); Albert Einstein College of Medicine (Bronx, NY); Albert Einstein College of Medicine (Bronx, NY); Albert Einstein College of Medicine (Bronx, NY); Albert Einstein College of Medicine (Bronx, NY); Albert Einstein College of Medicine (Bronx, NY); Albert Einstein College of Medicine (Bronx, NY); Albert Einstein College of Medicine (Bronx, NY); Albert Einstein College of Medicine (Bronx, NY); Albert Einstein College of Medicine (Bronx, NY); Albert Einstein College of Medicine (Bronx, NY); Albert Einstein College of Medicine (Bronx, NY); Albert Einstein College of Medicine (Bronx, NY); Albert Einstein College of Medicine (Bronx, NY); Albert Einstein College of Medicine (Bronx, NY); Albert Einstein College of Medicine (Bronx, NY); Albert Einstein College of Medicine (Bronx, NY); Albert Einstein College of Medicine (Bronx, NY); Albert Einstein College of Medicine (Bronx, NY); University of Pittsburgh (Pittsburgh, PA); University of Pittsburgh (Pittsburgh, PA); University of Pittsburgh (Pittsburgh, PA); University of Pittsburgh (Pittsburgh, PA); University of Pittsburgh (Pittsburgh, PA); University of Pittsburgh (Pittsburgh, PA); University of Pittsburgh (Pittsburgh, PA); University of Pittsburgh (Pittsburgh, PA); University of Pittsburgh (Pittsburgh, PA); University of Pittsburgh (Pittsburgh, PA); University of Pittsburgh (Pittsburgh, PA); University of Pittsburgh (Pittsburgh, PA); University of Pittsburgh (Pittsburgh, PA); University of Pittsburgh (Pittsburgh, PA); University of Pittsburgh (Pittsburgh, PA); University of Pittsburgh (Pittsburgh, PA); University of Pittsburgh (Pittsburgh, PA); University of Pittsburgh (Pittsburgh, PA); University of Pittsburgh (Pittsburgh, PA); University of Pittsburgh (Pittsburgh, PA); University of Pittsburgh (Pittsburgh, PA); University of Pittsburgh (Pittsburgh, PA); University of Pittsburgh (Pittsburgh, PA); University of Pittsburgh (Pittsburgh, PA); University of Pittsburgh (Pittsburgh, PA); University of Hawaii (Honolulu, HI); University of Hawaii (Honolulu, HI); University of Hawaii (Honolulu, HI); University of Hawaii (Honolulu, HI); University of Hawaii (Honolulu, HI); University of Hawaii (Honolulu, HI); University of Hawaii (Honolulu, HI); University of Hawaii (Honolulu, HI); University of Hawaii (Honolulu, HI); University of Hawaii (Honolulu, HI); University of Hawaii (Honolulu, HI); Southwest American Indian Centers (Phoenix, AZ; Shiprock, NM; Zuni, NM); Southwest American Indian Centers (Phoenix, AZ; Shiprock, NM; Zuni, NM); Southwest American Indian Centers (Phoenix, AZ; Shiprock, NM; Zuni, NM); Southwest American Indian Centers (Phoenix, AZ; Shiprock, NM; Zuni, NM); Southwest American Indian Centers (Phoenix, AZ; Shiprock, NM; Zuni, NM); Southwest American Indian Centers (Phoenix, AZ; Shiprock, NM; Zuni, NM); Southwest American Indian Centers (Phoenix, AZ; Shiprock, NM; Zuni, NM); Southwest American Indian Centers (Phoenix, AZ; Shiprock, NM; Zuni, NM); Southwest American Indian Centers (Phoenix, AZ; Shiprock, NM; Zuni, NM); Southwest American Indian Centers (Phoenix, AZ; Shiprock, NM; Zuni, NM); Southwest American Indian Centers (Phoenix, AZ; Shiprock, NM; Zuni, NM); Southwest American Indian Centers (Phoenix, AZ; Shiprock, NM; Zuni, NM); Southwest American Indian Centers (Phoenix, AZ; Shiprock, NM; Zuni, NM); Southwest American Indian Centers (Phoenix, AZ; Shiprock, NM; Zuni, NM); Southwest American Indian Centers (Phoenix, AZ; Shiprock, NM; Zuni, NM); Southwest American Indian Centers (Phoenix, AZ; Shiprock, NM; Zuni, NM); Southwest American Indian Centers (Phoenix, AZ; Shiprock, NM; Zuni, NM); Southwest American Indian Centers (Phoenix, AZ; Shiprock, NM; Zuni, NM); Southwest American Indian Centers (Phoenix, AZ; Shiprock, NM; Zuni, NM); Southwest American Indian Centers (Phoenix, AZ; Shiprock, NM; Zuni, NM); Southwest American Indian Centers (Phoenix, AZ; Shiprock, NM; Zuni, NM); Southwest American Indian Centers (Phoenix, AZ; Shiprock, NM; Zuni, NM); Southwest American Indian Centers (Phoenix, AZ; Shiprock, NM; Zuni, NM); Southwest American Indian Centers (Phoenix, AZ; Shiprock, NM; Zuni, NM); Southwest American Indian Centers (Phoenix, AZ; Shiprock, NM; Zuni, NM); Southwest American Indian Centers (Phoenix, AZ; Shiprock, NM; Zuni, NM); Southwest American Indian Centers (Phoenix, AZ; Shiprock, NM; Zuni, NM); Southwest American Indian Centers (Phoenix, AZ; Shiprock, NM; Zuni, NM); Southwest American Indian Centers (Phoenix, AZ; Shiprock, NM; Zuni, NM); Southwest American Indian Centers (Phoenix, AZ; Shiprock, NM; Zuni, NM); Southwest American Indian Centers (Phoenix, AZ; Shiprock, NM; Zuni, NM); Southwest American Indian Centers (Phoenix, AZ; Shiprock, NM; Zuni, NM); Southwest American Indian Centers (Phoenix, AZ; Shiprock, NM; Zuni, NM); Southwest American Indian Centers (Phoenix, AZ; Shiprock, NM; Zuni, NM); Southwest American Indian Centers (Phoenix, AZ; Shiprock, NM; Zuni, NM); Southwest American Indian Centers (Phoenix, AZ; Shiprock, NM; Zuni, NM); Southwest American Indian Centers (Phoenix, AZ; Shiprock, NM; Zuni, NM); Southwest American Indian Centers (Phoenix, AZ; Shiprock, NM; Zuni, NM); Southwest American Indian Centers (Phoenix, AZ; Shiprock, NM; Zuni, NM); Southwest American Indian Centers (Phoenix, AZ; Shiprock, NM; Zuni, NM); Southwest American Indian Centers (Phoenix, AZ; Shiprock, NM; Zuni, NM); Southwest American Indian Centers (Phoenix, AZ; Shiprock, NM; Zuni, NM); Southwest American Indian Centers (Phoenix, AZ; Shiprock, NM; Zuni, NM); Southwest American Indian Centers (Phoenix, AZ; Shiprock, NM; Zuni, NM); Southwest American Indian Centers (Phoenix, AZ; Shiprock, NM; Zuni, NM); Southwest American Indian Centers (Phoenix, AZ; Shiprock, NM; Zuni, NM); George Washington University Biostatistics Center (DPP Coordinating Center Rockville, MD); George Washington University Biostatistics Center (DPP Coordinating Center Rockville, MD); George Washington University Biostatistics Center (DPP Coordinating Center Rockville, MD); George Washington University Biostatistics Center (DPP Coordinating Center Rockville, MD); George Washington University Biostatistics Center (DPP Coordinating Center Rockville, MD); George Washington University Biostatistics Center (DPP Coordinating Center Rockville, MD); George Washington University Biostatistics Center (DPP Coordinating Center Rockville, MD); George Washington University Biostatistics Center (DPP Coordinating Center Rockville, MD); George Washington University Biostatistics Center (DPP Coordinating Center Rockville, MD); George Washington University Biostatistics Center (DPP Coordinating Center Rockville, MD); George Washington University Biostatistics Center (DPP Coordinating Center Rockville, MD); George Washington University Biostatistics Center (DPP Coordinating Center Rockville, MD); George Washington University Biostatistics Center (DPP Coordinating Center Rockville, MD); George Washington University Biostatistics Center (DPP Coordinating Center Rockville, MD); George Washington University Biostatistics Center (DPP Coordinating Center Rockville, MD); George Washington University Biostatistics Center (DPP Coordinating Center Rockville, MD); George Washington University Biostatistics Center (DPP Coordinating Center Rockville, MD); George Washington University Biostatistics Center (DPP Coordinating Center Rockville, MD); George Washington University Biostatistics Center (DPP Coordinating Center Rockville, MD); George Washington University Biostatistics Center (DPP Coordinating Center Rockville, MD); George Washington University Biostatistics Center (DPP Coordinating Center Rockville, MD); George Washington University Biostatistics Center (DPP Coordinating Center Rockville, MD); George Washington University Biostatistics Center (DPP Coordinating Center Rockville, MD); Central Biochemistry Laboratory (Seattle, WA); Central Biochemistry Laboratory (Seattle, WA); Central Biochemistry Laboratory (Seattle, WA); Carotid Ultrasound; CT Scan Reading Center; Epidemiological Cardiology Research Center- Epicare (Winston-Salem, NC); Epidemiological Cardiology Research Center- Epicare (Winston-Salem, NC); Epidemiological Cardiology Research Center- Epicare (Winston-Salem, NC); Epidemiological Cardiology Research Center- Epicare (Winston-Salem, NC); Epidemiological Cardiology Research Center- Epicare (Winston-Salem, NC); Epidemiological Cardiology Research Center- Epicare (Winston-Salem, NC); Epidemiological Cardiology Research Center- Epicare (Winston-Salem, NC); Epidemiological Cardiology Research Center- Epicare (Winston-Salem, NC); Epidemiological Cardiology Research Center- Epicare (Winston-Salem, NC); Epidemiological Cardiology Research Center- Epicare (Winston-Salem, NC); Nutrition Coding Center (Columbia, SC); Nutrition Coding Center (Columbia, SC); Quality of Well-Being Center (La Jolla, CA); Quality of Well-Being Center (La Jolla, CA); Quality of Well-Being Center (La Jolla, CA); NIH/NIDDK (Bethesda, MD); NIH/NIDDK (Bethesda, MD); NIH/NIDDK (Bethesda, MD); Centers for Disease Control & Prevention (Atlanta, GA); Centers for Disease Control & Prevention (Atlanta, GA); University of Michigan (Ann Arbor, MI); Massachusetts General Hospital, Boston, Massachusetts, United States of America; Broad Institute, Cambridge, Massachusetts, United States of America; Massachusetts General Hospital, Boston, Massachusetts, United States of America; Broad Institute, Cambridge, Massachusetts, United States of America; Broad Institute, Cambridge, Massachusetts, United States of America; Lund University, Lund, Sweden; Harvard School of Public Health, Boston, Massachusetts, United States of America; National Institute of Diabetes and Digestive and Kidney Diseases, National Institutes of Health, Bethesda, Maryland, United States of America; Coordinating Center, Millersville, Maryland, United States of America; National Institute of Diabetes and Digestive and Kidney Diseases, National Institutes of Health, Bethesda, Maryland, United States of America; Massachusetts General Hospital, Boston, Massachusetts, United States of America; Broad Institute, Cambridge, Massachusetts, United States of America; University of Maryland, College Park, Maryland, United States of America; University of Maryland, College Park, Maryland, United States of America; 1 Division of Endocrinology, Diabetes, and Nutrition, Department of Medicine, and Program in Genetics and Genomic Medicine, University of Maryland School of Medicine, Baltimore, Maryland, United States of America; 2 Division of Nephrology and Hypertension, Department of Medicine, Leonard M. Miller School of Medicine, University of Miami, Miami, Florida, United States of America; 3 The Biostatistics Center, George Washington University, Rockville, Maryland, United States of America; 4 Program in Medical and Population Genetics, Broad Institute of Harvard and MIT, Cambridge, Massachusetts, United States of America; 5 Division of Genetics, Brigham and Women's Hospital, Harvard Medical School, Boston, Massachusetts, United States of America; 6 Department of Medical Genetics, University Medical Center Utrecht, Utrecht, The Netherlands; 7 Department of Epidemiology, University Medical Center Utrecht, Utrecht, The Netherlands; 8 Center for Human Genetic Research, Department of Medicine, Massachusetts General Hospital, Boston, Massachusetts, United States of America; 9 Department of Medicine, Harvard Medical School, Boston, Massachusetts, United States of America; 10 Diabetes Research Center (Diabetes Unit), Department of Medicine, Massachusetts General Hospital, Boston, Massachusetts, United States of America; 11 Diabetes Clinic, Massachusetts General Hospital, Boston, Massachusetts, United States of America; 12 Department of Genetics, Harvard Medical School, Boston, Massachusetts, United States of America; 13 Geriatric Research and Education Clinical Center, Veterans Administration Medical Center, Baltimore, Maryland, United States of America; 14 Lipid Disorders Clinic, Division of Endocrinology, Diabetes, and Metabolism, Leonard M. Miller School of Medicine, University of Miami, Miami, Florida, United States of America; 15 The Diabetes Research Institute, Leonard M. Miller School of Medicine, University of Miami, Miami, Florida, United States of America; 16 Department of Medicine, Harvard Medical School, Boston, Massachusetts, United States of America; 17 Department of Clinical Sciences, Genetic and Molecular Epidemiology Unit, Lund University, Malmö, Sweden; 18 Department of Nutrition, Harvard School of Public Health, Boston, Massachusetts, United States of America; University of Alabama at Birmingham, United States of America

## Abstract

Weight-loss interventions generally improve lipid profiles and reduce cardiovascular disease risk, but effects are variable and may depend on genetic factors. We performed a genetic association analysis of data from 2,993 participants in the Diabetes Prevention Program to test the hypotheses that a genetic risk score (GRS) based on deleterious alleles at 32 lipid-associated single-nucleotide polymorphisms modifies the effects of lifestyle and/or metformin interventions on lipid levels and nuclear magnetic resonance (NMR) lipoprotein subfraction size and number. Twenty-three loci previously associated with fasting LDL-C, HDL-C, or triglycerides replicated (*P* = 0.04–1×10^−17^). Except for total HDL particles (r = −0.03, *P* = 0.26), all components of the lipid profile correlated with the GRS (partial |r| = 0.07–0.17, *P* = 5×10^−5^–1×10^−19^). The GRS was associated with higher baseline-adjusted 1-year LDL cholesterol levels (β = +0.87, SEE±0.22 mg/dl/allele, *P* = 8×10^−5^, *P*_interaction_ = 0.02) in the lifestyle intervention group, but not in the placebo (β = +0.20, SEE±0.22 mg/dl/allele, *P* = 0.35) or metformin (β = −0.03, SEE±0.22 mg/dl/allele, *P* = 0.90; *P*_interaction_ = 0.64) groups. Similarly, a higher GRS predicted a greater number of baseline-adjusted small LDL particles at 1 year in the lifestyle intervention arm (β = +0.30, SEE±0.012 ln nmol/L/allele, *P* = 0.01, *P*_interaction_ = 0.01) but not in the placebo (β = −0.002, SEE±0.008 ln nmol/L/allele, *P* = 0.74) or metformin (β = +0.013, SEE±0.008 nmol/L/allele, *P* = 0.12; *P*_interaction_ = 0.24) groups. Our findings suggest that a high genetic burden confers an adverse lipid profile and predicts attenuated response in LDL-C levels and small LDL particle number to dietary and physical activity interventions aimed at weight loss.

## Author Summary

The study included 2,993 participants from the Diabetes Prevention Program, a randomized clinical trial of intensive lifestyle intervention, metformin treatment, and placebo control. We examined associations between 32 gene variants that have been reproducibly associated with dyslipidemia and concentrations of lipids and NMR lipoprotein particle sizes and numbers. We also examined whether genetic background influences a person's response to cardioprotective interventions on lipid levels. Our analysis, which focused on determining whether common genetic variants impact the effects of cardioprotective interventions on lipid and lipoprotein particle size, shows that in persons with a high genetic risk score the benefit of intensive lifestyle intervention on LDL and small LDL particle levels is substantially diminished; this information may be informative for the targeted prevention of dyslipidemia, as it suggests that genetics might help identify persons in whom lifestyle intervention is likely to be an effective treatment for elevated lipids and lipoproteins. The NMR subfraction analyses provide novel insight into the biology of dyslipidemia by illustrating how numerous genetic variants that have previously been associated with lipid levels also modulate NMR lipoprotein particle sizes and number. This information may be informative for the targeted prevention of cardiovascular disease.

## Introduction

Dyslipidemia is a strong risk factor for atherosclerotic heart disease [1]–[3], has a well-defined genetic basis [4], and is modifiable through therapeutic lifestyle changes and weight-loss interventions [5], [6]. Individuals at risk for diabetes are also at high risk of cardiovascular disease [7], and individualized lifestyle intervention programs, like the one incorporated into the Diabetes Prevention Program (DPP), have a salutary effect on dyslipidemia and cardiovascular disease risk in this population. However, the cost of widespread implementation of such interventions has been highlighted as a major limitation [8] and not all benefit equally from such interventions. Identifying persons most likely to benefit from intensive lifestyle modification could provide justification for targeting this subpopulation first, making the clinical translation of findings from studies such as the DPP more feasible.

Selection of persons whose dyslipidemia is likely to respond well to lifestyle interventions or pharmacotherapy could help target resources and optimize prevention strategies. To do so requires knowledge of the underlying risk factors for the trait and knowledge of how personal characteristics interact with exercise, diet, and weight loss. Although the heritability of polygenic dyslipidemia [9]–[11] and its sequelae [12] have been elucidated, little is known of how lifestyle interventions modify the effects of these loci, singly or in combination, on lipid profiles. Thus, learning how a person's genetic background modulates his or her response to therapeutic lifestyle changes and weight-loss interventions might help optimize the targeting of interventions designed to mitigate cardiovascular and metabolic disease risk.

The purpose of this study was to examine whether loci reliably associated with polygenic dyslipidemia modified the response to cardio-protective interventions in the DPP, a randomized clinical trial of intensive lifestyle modification, metformin treatment, or placebo with standard care. We hypothesized i) that the baseline lipid profiles of DPP participants would be associated with gene variants known to associate with polygenic dyslipidemia and ii) that improvement in lipidemia following treatment would depend on these same genetic variants. We also used NMR spectroscopy to characterize the associations of these previously reported loci with lipoprotein subfractions.

## Results

Table 1 shows participant characteristics stratified by DPP treatment arm. The effects of the DPP interventions on 1 yr changes in weight [14], insulin secretion [13], beta-cell function [13], and lipid traits [29] are reported in detail elsewhere.

**Table 1:** Baseline Characteristics of the Study Population by Treatment Group [Quantitative Traits Are Shown as Median (Interquartile Range)].

Trait	Placebo	Metformin	Lifestyle
n	947	939	962
Age	49 (43–57)	50 (44–57)	49 (42–58)
Sex [M∶F (% male)]	290∶657 (31% male)	321∶618 (34% male)	308∶654 (32% male)
White/AA/Hisp/Asian/AI: *n* (%)	515(54)/207(22)/157(17)/38(4)/30(3)	534(57)/194(21)/155(17)/33(4)/23(3)	517(54)/191(20)/173(18)/53(6)/28(3)
BMI (kg/m^2^)	33.4 (29.2–38.3)	33.0 (29.1–37.7)	32.8 (29.0–37.3)
Waist Circumference (cm)	104.4 (95–114.7)	104.3 (94.7–114.0)	103.8 (95.0–113.6)
Total Cholesterol (mg/dl)	201 (178–227)	202 (177–225)	202 (179–227)
LDL-C (mg/dl)	123 (102–147)	123 (103–145)	124 (102–145)
HDL-C (mg/dl)	43 (37–50)	44 (38–52)	44 (37–53)
Triglycerides (mg/dl)	146 (102.5–205.5)	135 (97–195)	136 (94–200)
Large HDL particles (umol/L)	3.3 (2.1–5.5)	3.4 (2.2–5.3)	3.3 (2.2–5.4)
Small HDL particles (umol/L)	19 (16.1–22.2)	19.2 (16.3–22.6)	18.9 (15.8–21.8)
Total HDL particles (umol/L)	34.1 (30.4–38.5)	34.7 (31.2–38.9)	33.95 (30.2–38.1)
HDL size (nm)	8.8 (8.6–9.1)	8.8 (8.6–9.1)	8.8 (8.6–9.1)
LDL size (nm)	0.263 (0.237–0.289)	0.263 (0.237–0.289)	0.263 (0.237–0.289)
Total LDL particles (nmol/L)	1369 (1140–1629)	1367 (1108–1607)	1332 (1123–1591)
Small LDL particles (nmol/L)	788 (517–1059)	779 (525–1041)	764 (520–1040)
Total VLDL particles (nmol/L)	63.3 (43.9–88.1)	62.4 (42.6–86.2)	63.2 (42.1–88.5)
Large VLDL particles (nmol/L)	5.4 (2.8–10.8)	6.1 (2.8–11)	5.9 (2.7–10.8)
VLDL size (nm)	52.2 (47.0–58.9)	53.0 (46.9–59.4)	52.8 (47.0–59.0)

### Individual SNP Replication

Thirty-two SNPs previously associated with triglycerides (TG), low-density lipoprotein-cholesterol (LDL-C) and/or high-density lipoprotein-cholesterol (HDL-C) levels were considered [10]. Thirty-one of these were successfully genotyped in the DPP, and two SNPs in *CETP*, serving as HapMap proxies (r^2^≥0.90) for rs173539, including rs247616, were subsequently successfully genotyped, with rs247616 retained as the replacement for rs173539. Twenty-three of these 32 non-redundant SNPs replicated with their respective traits in a directionally consistent manner (*P*≤0.05), including 8/11 for TG, 9/14 for HDL-C and 8/11 for LDL-C. Two of the SNPs, rs12678919 and rs964184, replicated for both HDL-C and TG (Table S1).

### Association of Individual SNPs with All Four Lipid Traits and Ten Lipoprotein Traits

Additionally, we evaluated the associations of the 32 lipid loci with baseline lipids and nuclear magnetic resonance (NMR)-derived lipoprotein traits (Large HDL particles, Small HDL particles, Total HDL particles, HDL size, LDL size, Total LDL particles, Small LDL particles, Total VLDL particles, Large VLDL particles, VLDL size). Of all analyses of baseline traits, roughly one third of the tests were nominally significant associations, and 35 associations were significant after correcting for all 448 hypothesis tests; these involved 12 SNPs and 13 traits (Table S2). Interestingly, SNP rs10401969 did not replicate for LDL-C (C vs. T: β±SEM = −0.1±1.6 mg/dl, additive *P* = 0.94), but was associated with decreased large VLDL (mean 5.43, 4.26, 4.07 nmol/L for TT, TC, CC genotypes respectively, additive *P* = 4×10^−5^) and smaller VLDL size (53.18, 50.94, 49.55 nm, *P* = 2×10^−6^). SNP rs7679 did not quite reach nominal significance for decreased HDL-C (C vs. T: β ±SEM = −0.016±0.009 ln mg/dl, additive *P* = 0.07), but was very strongly associated with increased small HDL particle number (17.93, 20.14 and 21.89 µmol/L for TT, CT and CC genotypes respectively; additive *P* = 4×10^−18^) and consequently total HDL particle number (34.07, 35.10 and 36.78 µmol/L, *P* = 2×10^−5^).

### Association of Genetic Risk Score (GRS) with Baseline Lipid and Lipoprotein Traits

A lipid GRS was calculated for each individual by first replacing missing genotypes with ethnicity-specific imputed means and then adding up the number of risk alleles possessed for each of the 32 independent SNPs. Of the 32 SNPs evaluated, 11 were originally associated in the meta-analysis with LDL cholesterol, 10 with HDL cholesterol only, seven with triglycerides only, and four with both HDL cholesterol and triglycerides. A risk allele was defined as one associated with increased TG or LDL-C or decreased HDL in the original meta-analysis [10]. After adjustment for age, sex, ethnicity, and BMI, the GRS was significantly associated with all baseline traits evaluated except total HDL particles (*P* = 0.26, Table 2). The following are *P*-values for the effects of the GRS, as a quantitative covariate, and geometric means for the upper and lower ethnicity-specific GRS quartiles for each trait. A higher GRS was associated with elevated baseline levels of: total cholesterol (*P* = ×410^−11^, 206 vs. 195 mg/dl), LDL-C (*P* = ×910^−8^;, 129 vs. 121 mg/dl arithmetic means), TG (*P* = ×410^−19^, 160 vs. 127 mg/dl), total VLDL particles (*P* = ×610^−14^, 67 vs. 53 nmol/L), large VLDL particles (*P* = ×110^−14^, 6.57 vs. 4.21 nmol/L), total LDL particles (*P* = ×210^−10^, 1412 vs. 1262 nmol/L), small LDL particles (*P* = ×210^−11^, 743 vs. 543 nmol/L), small HDL particles (*P* = µ0.0005, 19.17 vs. 18.10 mol/L), and VLDL particle size (*P* = ×110^−5^, 53.86 vs. 51.84 nm). A higher GRS was also associated with lower baseline levels of: HDL-C (*P* = ×110^−15^, 43 vs. 47 mg/dl), LDL particle size (*P* = ×110^−19^, 0.256 vs. 0.269 nm), large HDL particles (*P* = ×210^−8^µ, 2.98 vs. 3.68 mol/L), and HDL particle size (*P* = 0.0003, 8.82 vs. 8.90 nm). All of these results are consistent with a greater number of risk alleles increasing the atherogenicity of the lipoprotein profile.

**Table 2:** Association of 32-SNP GRS with Baseline Lipid and Lipoprotein Traits (n2,843).

Trait	Q1	Q2	Q3	Q4	%diff**	Partial r*	±BetaSE/unit	p-value***
	–(2233*)	–(3335*)	–(3537*)	–(3744*)				
n	875	751	636	586	–	**–**	–	**–**
Chol (mg/dl)	–195 (192197)	–199 (196202)	–201 (199204)	–206 (204209)	%6	0.12	+±0.0070.001	**×410^−11^**
LDL-C (mg/dl)	–121 (119123)	–124 (122127)	–126 (124129)	–129 (127132)	%7	0.11	+±1.010.19	**×910^−8^**
HDL-C (mg/dl)	–47 (4748)	–46 (4546)	–45 (4446)	–43 (4244)	%9	−0.15	−±0.0110.001	**×110^−15^**
TG (mg/dl)	–127 (123131)	–140 (135145)	–146 (141152)	–160 (153166)	%26	0.14	+±0.0260.003	**×410^−19^**
LDL size (nm)	–0.269 (0.2670.271)	–0.264 (0.2620.266)	–0.260 (0.2580.262)	–0.256 (0.2540.258)	%5	−0.17	−±0.00590.0007	**×110^−19^**
Total VLDL particles (nmol/L)	–53 (5155)	–59 (5661)	–62 (5965)	–67 (6471)	%26	0.17	+±0.0270.004	**×610^−14^**
Large VLDL particles (nmol/L)	–4.21 (3.914.54)	–4.93 (4.545.35)	–5.87 (5.376.42)	–6.57 (5.997.2)	%56	0.15	+±0.0520.057	**×110^−14^**
Total LDL particles (nmol/L)	–1262 (12341292)	–1304 (12721337)	–1345 (13081382)	–1412 (13731453)	%12	0.13	+±0.0130.002	**×210^−10^**
Small LDL particles (nmol/L)	–543 (511577)	–621 (581664)	–703 (654756)	–743 (689801)	%37	0.17	+±0.0370.005	**×210^−11^**
µLarge HDL particles (mol/L)	–3.68 (3.523.86)	–3.31 (3.153.48)	–3.21 (3.043.39)	–2.98 (2.823.15)	%19	−0.15	−±0.0230.004	**×210^−8^**
µSmall HDL particles (mol/L)	–18.10 (17.7118.49)	–18.42 (17.9918.85)	–18.57 (18.119.05)	–19.17 (18.6719.69)	%6	0.08	+±0.0070.002	**0.0005**
HDL size (nm)	–8.90 (8.878.93)	–8.87 (8.838.9)	–8.83 (8.88.87)	–8.82 (8.788.85)	%1	−0.07	−±0.00110.0003	**0.0003**
VLDL size (nm)	–51.84 (51.2152.48)	–52.34 (51.6653.04)	–53.65 (52.8854.43)	–53.86 (53.0754.66)	%4	0.10	+±00470.0011	**×110^−5^**
µTotal HDL particles (mol/L)	–34.62 (34.1735.07)	–34.19 (33.7134.68)	–34.52 (33.9935.06)	–34.17 (33.6334.73)	%1	−0.03	−±0.00130.0012	0.26

* %%Quartiles were assigned separately in each ethnic group, leading to slight overlap in quartile ranges of number of risk alleles. Traits are age-, sex-, ethnicity- and BMI-adjusted geometric means and 95 confidence intervals, except LDL-C, for which arithmetic mean and 95 confidence interval are shown. Ethnic specific quartile upper limits are 32, 34, 36, 44 alleles for both Whites and African Americans, 32, 35, 37, 43 alleles for Hispanics, 32, 35, 37, 42 alleles for Asians/Pacific Islanders and 33, 34, 36, 40 alleles for American Indians.

** Percent difference between Q4 and Q1 in reference to Q1.

*** Partial r and p-value based on analysis of GRS as a quantitative covariate with adjustment for age, sex, ethnicity and BMI.

### GRS×Intervention Interactions of Baseline-Adjusted One-Year Traits

**Figure 1:**
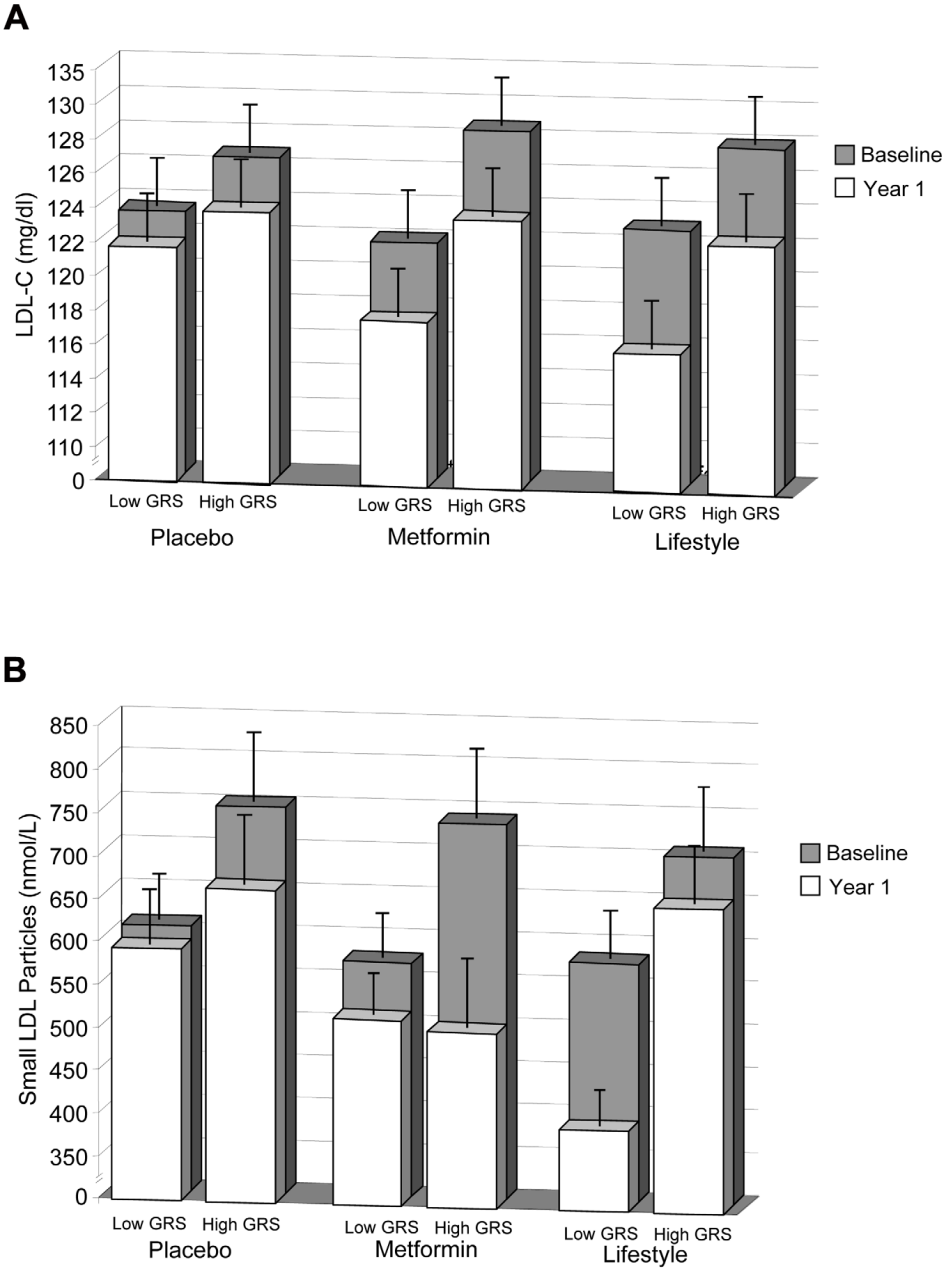
LDL-C levels. LDL-C levels at baseline and 1 year (A) and small LDL particle levels at baseline and 1 year (B) stratified by treatment group and lipid GRS. Each column shows ethnicity-adjusted arithmetic (for LDL-C) or geometric (for small LDL particles) means (with upper 95 confidence), stratified above and below (less than or equal to) the ethnic-specific median GRS value. Ethnic-specific median GRS values are 34 alleles for Caucasian, African American and American Indian ethnicities and 35 for Hispanic and Asian/Pacific Islander ethnicities.

Two traits showed evidence of GRS×lifestyle interaction: LDL-C (*P* = 0.02) and small LDL particles (*P* = 0.01, Table 3; Figure 1; Figure S1a–S1f). For these two traits, there was a residual detrimental impact of GRS in the lifestyle (i.e., the GRS was associated with higher levels at one year even after adjusting for baseline levels) but not the metformin or placebo group, suggesting that the lifestyle intervention was less effective at lipid-lowering in those with a higher genetic burden. A unit (allele) GRS increase was associated with higher residual LDL-C levels in the lifestyle group (*β*+±0.087, SEE0.022 mg/dl, *P* = ×810^−5^) but not in the metformin (*β*−±0.03, SEE0.22 mg/dl, *P* = 0.90) or placebo (*β*+±0.20, SEE0.22 mg/dl, *P* = 0.35) groups (Figure 1). Similarly, the GRS was associated with higher residual ln-small LDL particles in the lifestyle group (*β*+±0.030, SEE0.0.012 ln nmol/L, *P* = 0.01), but not in the metformin (*β*−±0.013, SEE0.008 ln nm/L, *P* = 0.12) or placebo (*β*−±0.002, SEE0.008, *P* = 0.74) groups (Figure 1×). There were no metforminGRS interactions significant at the *P* = ×0.05 level. In addition to the three traits discussed, several traits showed residual detrimental effects of the GRS in one or more strata (total cholesterol, TG, LDL size, total VLDL particles, large HDL particles, and HDL size) without any statistical evidence of treatmentGRS interaction (Table 3).

**Table 3:** Association of 32-SNP GRS with Baseline-Adjusted One-Year Lipid and Lipoprotein Traits (n2,686).

Trait	Placebo	Metformin	p	ILS	p
	β_GRS_±SE	β_GRS_±SE	GRSxMet	β_GRS_±SE	×GRSILS
n	895	884	–	907	–
Chol (mg/dl)	+±0.0010.001	−±0.00020.0012	0.42	***+±0.0040.001***	0.09
LDL-C (mg/dl)	+±0.200.22	−±0.030.22	0.64	***+±0.870.22***	**0.02**
HDL-C (mg/dl)	−±0.0020.001	−±0.0010.001	0.78	−±0.0010.002	0.61
TG (mg/dl)	**+±0.0080.004**	+±0.0040.004	0.50	+±0.0050.004	0.59
LDL size (nm)	−±0.00040.0009	**−±0.00220.0009**	0.17	***−±0.00240.0009***	0.22
Total VLDL particles (nmol/L)	+±0.00030.0058	+±0.00770.0054	0.49	**+±0.01420.0059**	0.12
Large VLDL particles (nmol/L)	−±−0.00360.0096	+±0.01060.0106	0.46	+±0.00850.0123	0.61
Total LDL particles (nmol/L)	−±0.00280.0027	+±0.00130.0027	0.34	+±0.00350.0030	0.10
µLarge HDL particles (mol/L)	**−±0.0140.006**	**−±0.0110.006**	0.90	−±0.0090.006	0.89
µSmall HDL particles (mol/L)	−±0.0010.003	+±0.0010.003	0.56	+±0.0010.006	0.45
Small LDL particles (nmol/L)	−±0.0020.008	+±0.0130.008	0.24	***+±0.0300.012***	**0.01**
HDL size (nm)	−±0.00040.0004	**−±0.00090.0004**	0.38	**−±0.00110.0005**	0.16
VLDL size (nm)	−±0.000050.00165	+±0.000150.00169	0.97	−±0.000960.00189	0.54
µTotal HDL particles (mol/L)	−±0.00030.0015	−±0.00210.0016	0.36	−±0.00100.0017	0.76

All traits except LDL-C ln-transformed prior to analysis and presentation of beta coefficients and standard errors. Treatment-specific results in **bold**≤; indicate p0.05 ***bold italics***<; indicates p0.01 ***underlined bold italics***< indicate p0.001.

## Discussion

In the present study, the majority of previously associated SNPs replicated for baseline lipid traits, and there was a statistically significant relationship between the GRS and the vast majority of standard lipid traits and NMR lipoprotein subfractions. Importantly, in several cases the evidence for association with NMR subfractions was much stronger than for the original standard lipid trait, which may be owing to the relative proximity of the subfractions to the genetic loci. For example, SNP rs7679, originally associated with total HDLC levels in previous GWASs, in the DPP was not significantly associated with HDL-C (*P* = 0.07) but was strongly associated with small HDL particle levels (*P* = ×410^−18^). This SNP is near *PLTP*, encoding phospholipid transfer protein, a molecule directly influencing HDL particle size [14]. Such findings extend our understanding of lipid biology and suggest that, compared to standard lipid levels, measurements of lipoprotein subfractions may provide a more effective way of capturing genetically influenced risk. This is particularly important, as recent studies have shown that HDL-C is a heterogeneous trait, which in the context of clinical use may benefit from sub-stratification by genotype [15].

We also observed that within the lifestyle group but not the placebo or metformin groups, the GRS was associated with higher LDL-C and small LDL particle levels after one year of intervention. These findings suggest that the genetic burden on these traits cannot be completely overcome by lifestyle modification. However, even those with the greatest genetic burden benefit to a limited extent from lifestyle intervention in terms of LDL-C reduction (Figure 1A), although the effect on LDL particle size reduction is almost completely ablated (Figure 1B'). Even a true residual effect of lifestyle on LDL-C in people with the highest GRS does not negate the clinical relevance of our findings in terms of potential to facilitate tailored treatment decisions. Seeing less of an effect of lifestyle in a particular patient subgroup indicates that these persons may benefit from more frequent surveillance, more intense lifestyle interventions, or aggressive pharmaceutical interventions to supplement lifestyle interventions. Conversely, knowing that lifestyle intervention is likely to be adequate in persons with the lowest genetic burden may maximize the patients diet adherence and potentially reduce the costs and side effects associated with prescribing lipid-lowering medications unnecessarily. The availability of information on genetic background may also facilitate patient-provider dialogue, owing to improved diagnostic accuracy. This is similar to the strategy used to control cholesterol levels in patients with a monogenic disorder such as familial hypercholesterolemia due to a severe loss of function mutation in LDLR, where lifestyle intervention combined with pharmacotherapy is needed to bring LDL-C levels within an acceptable range (Third Report of the NCEP-ATP III on the Detection, Evaluation, and Treatment of High Blood Cholesterol in Adults).

×It is also important to bear in mind that the interaction effects may be underestimated in our paper. This is because the majority of SNPs included in the GRS are likely to be imperfect proxies for unobserved functional variants, resulting in some degree of genotype misclassification. Moreover, all 32 SNPs were included within the GRS, even though not all SNPs convey statistically significant effects in the DPP and do not individually modify the effects of the interventions. A parsimonious GRS including only those SNPs that are statistically significant in the DPP would likely be overfitted to our data, resulting in biased conclusions about the strength and magnitude of genetreatment interactions.

Dyslipidemia is a long-established risk factor for CVD [1]–[3]. Thus, the primary and secondary prevention of atherosclerotic CVD often involves intervening on lipid levels [16]. Lifestyle interventions [17] and metformin treatment [18]; that result in weight loss have the potential to improve lipid profiles nevertheless, as long recognized [19], changes in lipid profiles following interventions vary greatly from one person to the next. Some of the variability in response to interventions may be because genotypes modulate the effects of preventive interventions on lipid homeostasis and CVD risk [20].

Of the many known dyslipidemia-predisposing loci discovered so far [10]×, only a handful have been the focus of studies testing hypotheses of genetreatment interactions [21]–[28], and most of these studies are small (*N*<150), non-randomized trials of dietary intervention. Although some of these studies have focused on genomic regions that are confirmed to harbor dyslipidemia-predisposing loci, such as *APOB*, *CETP*, *LIPC* and *LPL* [21], [22], [24]–[28], no exhaustive studies testing whether GWAS-discovered loci [10], [29], [30] modify response to treatments have been previously reported.

The GRS used in this study attenuated the impact of the DPP lifestyle intervention on LDL levels and small LDL particle number. This suggests that a genetic predisposition to high LDL levels and more small LDL particles is difficult to overcome through lifestyle intervention alone. These data also unmask the effects of an underlying genetic defect of LDL levels and small LDL particles found in individuals with a high genetic burden, which becomes visible when adiposity and blood TG content are reduced through lifestyle intervention. This information may justify the combination of lifestyle and lipid lowering drug treatment from the outset in these individuals, rather than the usual approach of stepping from lifestyle to drug therapy when the former fails.

;The DPP lifestyle intervention prioritized weight loss, daily fat gram intake and physical activity goals over intake of saturated fat, cholesterol, viscous fiber and plant stanols/sterols this may have influenced the nature of the changes in the lipid profile. When compared to the metformin and placebo groups, the lifestyle intervention group reported improved physical activity levels and reductions in calorie intake, resulting in significantly greater weight losses [31], each of which has major influences on TG levels. The lifestyle intervention group reported significantly greater reductions in percent calories from total fat and saturated fat than the metformin and placebo groups [31]. However, they did not, on average, achieve the National Cholesterol Education Program target for saturated fat intake and did not focus on the other therapeutic lifestyle changes, such as the additional dietary changes mentioned above, that often have the largest effects on LDL concentrations. The ethnic diversity of the DPP cohort facilitates the generalizability of results, but may also lead to confounding by population stratification in genetic analyses. However, Sensitivity analyses in the European White sub-cohort of the DPP yielded comparable effect estimates to the results obtained in the entire DPP genetics cohort (results for baseline traits shown in Table S3), supporting the conclusion that confounding by population stratification is unlikely to explain our findings.

×Interestingly, no significant interaction was observed between the GRS and other biochemical components of the lipid profile in the present study. It is important to bear in mind, however, that despite being the largest clinical trial of its kind, the DPP is only moderately powered to detect genetreatment interactions [32];× it is likely, therefore, that genetreatment interactions that are small in magnitude will have been overlooked here. Moreover, during the course of writing this paper, many smaller impact lipid loci have been discovered [9], [11]. Thus, it is possible that with a larger sample size and the inclusion of some or all of these additional loci, we may have discovered interaction effects on other lipid traits.

In summary, we have shown that common genetic loci that influence polygenic dyslipidemia also modify the effects of clinical interventions designed to mitigate cardiovascular and metabolic risk. This report is the first comprehensive effort to examine validated lipid loci within the context of a large randomized clinical trial. The findings of this study may facilitate the implementation of complex trait genetics into the clinical setting.

## Methods

### Participants

The DPP was a multi-center randomized controlled trial that examined the effects of metformin or intensive lifestyle modification on the incidence of type 2 diabetes [33], [34]∼∼%%∼– = . Briefly, overweight persons with elevated but non-diabetic fasting and post-challenge glucose levels were randomized to receive placebo, metformin (850 mg twice daily) or a program of intensive lifestyle modification. The lifestyle intervention was designed to achieve 150 min/wk of physical activity and 7 weight loss via focus on daily fat gram goals. Fat gram goals were based on initial weight and 25 of calories from fat using a calorie level estimate to produce a weight loss of 0.51 kg/wk. The principal endpoint was the development of diabetes by confirmed semi-annual fasting plasma glucose or annual oral glucose tolerance testing (OGTT). Other phenotypes, such as changes in weight, waist circumference, lipids, insulin and glucose, were also ascertained. Written, informed consent was obtained from each participant, and each of the 27 DPP centers obtained institutional review board approval prior to initiation of the study protocol. A total of 2,993 participants in the placebo, lifestyle and metformin groups had DNA available and provided consent for genetic analysis. Individuals taking lipid lowering medications at baseline (n145) were excluded from all analyses.

### Measurements

≥[]All participants fasted for 12 hrs the night before blood was drawn from an antecubital vein. Standard blood lipid measurements (triglyceride TG, total cholesterol, HDL-C, calculated LDL-C) were performed at the DPP central biochemistry laboratory. TG and total cholesterol levels were measured using enzymatic methods standardized to the Centers for Disease Control and Prevention reference methods [35]. HDL fractions for cholesterol analysis were obtained by the treatment of whole plasma with dextran sulfate Mg^+2^ [36]. LDL cholesterol was calculated by the Friedewald equation [37]>β. In participants with TGs4.5 mmol/l, the lipoprotein fractions were separated using preparative ultracentrifugation of plasma by quantification [38]. Lipoprotein subclass particle concentrations and average VLDL, LDL, and HDL particle diameters were measured by NMR spectroscopy at LipoScience, Inc (Raleigh, NC) with modification of existing methods [39].

### Genotyping

Thirty-two SNPs previously associated with lipid concentrations in GWAS meta-analyses [10] were selected. DNA was extracted from peripheral blood leukocytes using standard methods. Genotyping was performed by allele-specific primer extension of multiplex amplified products and detection using matrix-assisted laser desorption ionization time-of-flight mass spectrometry on a Sequenom iPLEX platform [40]%%. The mean genotyping success rate was 96.7. The minimum call rate was 94.0. All SNPs were in Hardy-Weinberg equilibrium within each self-reported ethnic group.

### Statistical Analysis

The SAS software v9.2 (SAS, Carey, NC) was used for analyses. Baseline total cholesterol, HDL-C, TG and all lipoprotein sub-fraction levels were natural log transformed for non-normality, and LDL-C was evaluated directly. For replicating the previously reported associations of SNPs with baseline traits and evaluating the association of the individual SNPs with NMR lipoprotein particle sizes and numbers, measurements were compared across genotypic groups by ANCOVA (general model, 2 df *F* test for three possible genotypes), and evidence for an additive effect of genotype was also evaluated using the measured genotype approach, in which each genotype was assigned a value of 0, 1 or 2 according to the number of minor alleles. Analyses of baseline traits were adjusted for age, sex, self-reported ethnicity (to minimize confounding due to potential differences in both allele frequency and lipid traits across ethnicities) and BMI. For the individual SNP analyses, the Bonferroni-corrected *P*-value for significance was set at *P*<× = ; = 0.0001 to account for multiple comparisons (32 SNPs14 traits448 tests 0.05/4480.0001).

A genetic risk score (GRS) was calculated from the 32 SNPs using the direction of association from the initial association seen in the published meta-analysis [10]; for each SNP, an allele was designated as a risk allele if it was associated with higher TG or LDL-C and/or lower HDL-C. In order to be able to incorporate all individuals in the analysis, including those missing genotypes at one or more loci, a simple imputation procedure within each self-reported ethnic group was implemented (in order to account for allele frequency differences across ethnicities) prior to score calculation. First, after coding the genotype as the number of minor alleles (0, 1 or 2), an ethnicity-specific mean genotype was calculated and rounded to the nearest whole number. Missing genotypes were replaced by the appropriate rounded mean genotype [41]%××. We calculated a GRS for each individual by adding up the number of risk alleles for each of the 32 tested SNPs, where a risk allele was defined as one associated with increased TG or LDL-C or decreased HDL. The GRS was then included as a quantitative independent variable in a multiple regression model for each baseline lipid/lipoprotein trait to test for association, adjusted for age, sex, self-reported ethnicity, and BMI. GRS quartiles were constructed separately within each self-reported ethnicity prior to calculating quartile-specific arithmetic means or geometric means and 95 confidence intervals. To test for interaction of the risk score with treatment, a multiple regression model was constructed with the 1 year value as the outcome variable and including GRS, lifestyle and metformin treatment and GRSlifestyle and GRSmetformin terms, along with adjustments for the corresponding baseline trait, baseline age, sex and self-reported ethnicity.

### Sample Size and Power

*A priori* power calculations are an important study-planning tool, providing relevant information on likely effect sizes and variances is accessible. It is possible to obtain a broad understanding of the power constraints of our study by extrapolating results from other experimental settings (as described in detail in [42]), but specific *a priori*× power calculations could not be performed for the current study because reliable effect estimates and variances for tests of genetreatment interactions for the index genotypes and phenotypes were unavailable in the published literature at the time this study was planned. *Post-hoc*' power calculations were not performed, as these are well known to cause bias when interpreting a studys results [43]–[46]. However, confidence intervals are included in the figures, which give insight into the precision of the GRS effect estimates and hence the power to detect those estimates in the DPP cohort.

## Supporting Information

Figure S1 a: Box plots for Small LDL particles measured at baseline in the placebo arm of the Diabetes Prevention Program stratified by level of the genetic risk score. b: Box plots for Small LDL particles measured at baseline in the metformin arm of the Diabetes Prevention Program stratified by level of the genetic risk score. c: Box plots for Small LDL particles measured at baseline in the lifestyle arm of the Diabetes Prevention Program stratified by level of the genetic risk score. d: Box plots for Small LDL particles measured at 1 yr follow-up in the placebo arm of the Diabetes Prevention Program stratified by level of the genetic risk score. e: Box plots for Small LDL particles measured at 1 yr follow-up in the metformin arm of the Diabetes Prevention Program stratified by level of the genetic risk score. f: Box plots for Small LDL particles measured at 1 yr follow-up in the lifestyle arm of the Diabetes Prevention Program stratified by level of the genetic risk score.
(PPTX)

Table S1 ≤Details of individual SNP replication results (n2,843). The table compares results for each of the SNP loci in published meta-analysis and elsewhere with those reported here in the DPP. Analyses and means adjusted for age, sex, ethnicity and BMI.
(DOCX)

Table S2 <Associations of individual SNPs with lipid or lipoprotein traits significant at the Bonferroni-significant p-value of 0.0001 for additive model (see Table 1 for units). Shown are geometric means for all traits except LDL-C, for which arithmetic means are shown. All analyses and means adjusted for age, sex, ethnicity and BMI.
(DOCX)

Table S3  = Associations of individual SNPs with lipid or lipoprotein traits measured at baseline in White participants from the DPP (n1,565). Analyses were performed to determine whether population stratification owing to the mutliethnic nature of the DPP is likely to confound the associations reported in the main analyses. The comparability of the results in White DPP participants with the main analyses indicates that confoudning by population stratification is unlikely to underly the main results reported here. Analyses and means adjusted for age, sex, and BMI.
(DOCX)
