# Supplementary material for: Genetic Modulation of Lipid Profiles following Lifestyle Modification or Metformin Treatment: The Diabetes Prevention Program
Source: PLoS Genet. 2012 Aug 30;8(8):e1002895. doi: 10.1371/journal.pgen.1002895 (PMC3431328; doi:10.1371/journal.pgen.1002895)
Supplement: Figure S1 — a: Box plots for Small LDL particles measured at baseline in the placebo arm of the Diabetes Prevention Program stratified by level of the genetic risk score. b: Box plots for Small LDL particles measured at baseline in the metformin arm of the Diabetes Prevention Program stratified by level of the genetic risk score. c: Box plots for Small LDL particles measured at baseline in the lifestyle arm of the Diabetes Prevention Program stratified by level of the genetic risk score. d: Box plots for Small LDL particles measured at 1 yr follow-up in the placebo arm of the Diabetes Prevention Program stratified by level of the genetic risk score. e: Box plots for Small LDL particles measured at 1 yr follow-up in the metformin arm of the Diabetes Prevention Program stratified by level of the genetic risk score. f: Box plots for Small LDL particles measured at 1 yr follow-up in the lifestyle arm of the Diabetes Prevention Program stratified by level of the genetic risk score. (PPTX) [file pgen.1002895.s001.pptx]

## Slide 1
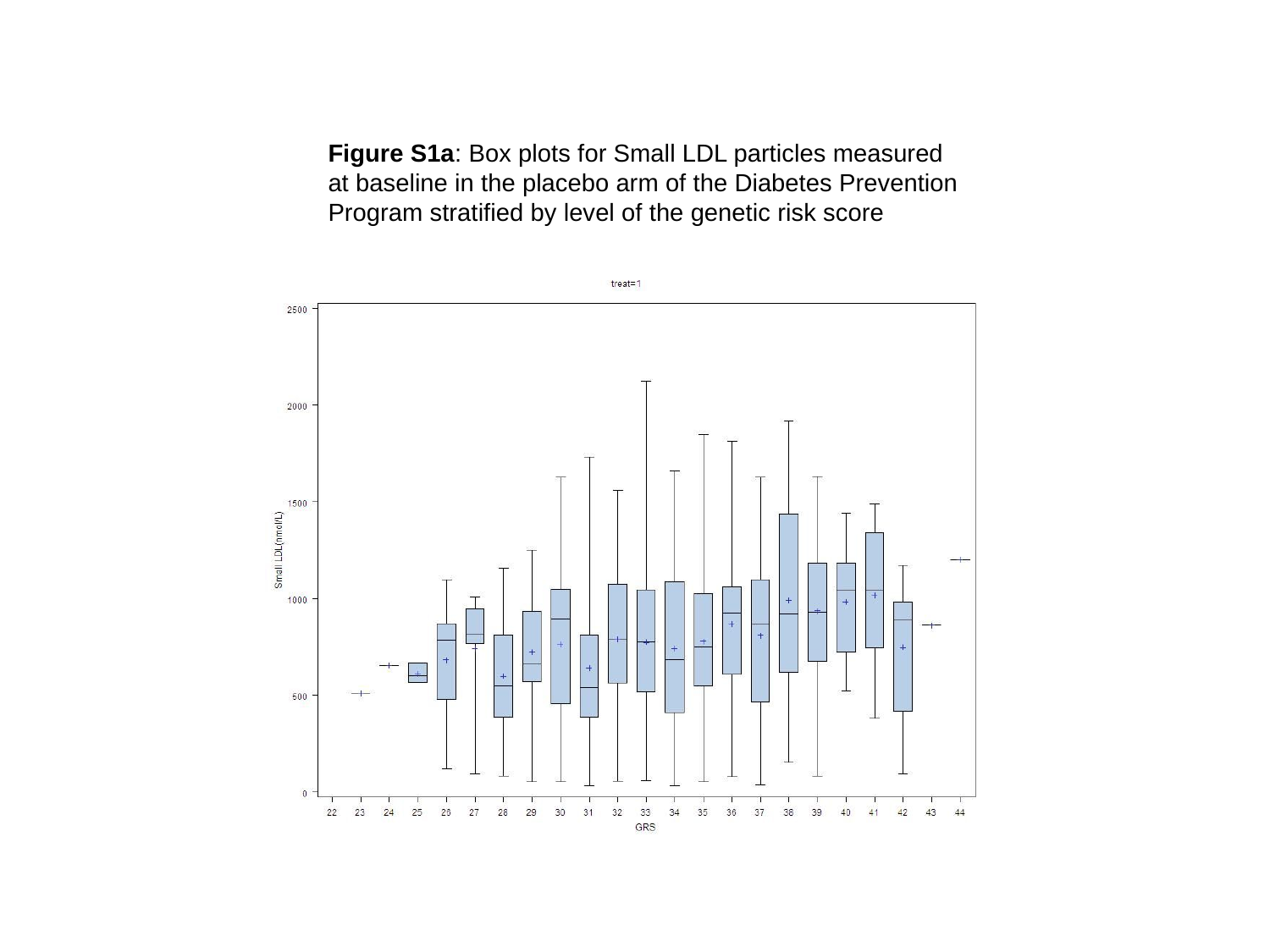

# Figure S1a: Box plots for Small LDL particles measured at baseline in the placebo arm of the Diabetes Prevention Program stratified by level of the genetic risk score

## Slide 2
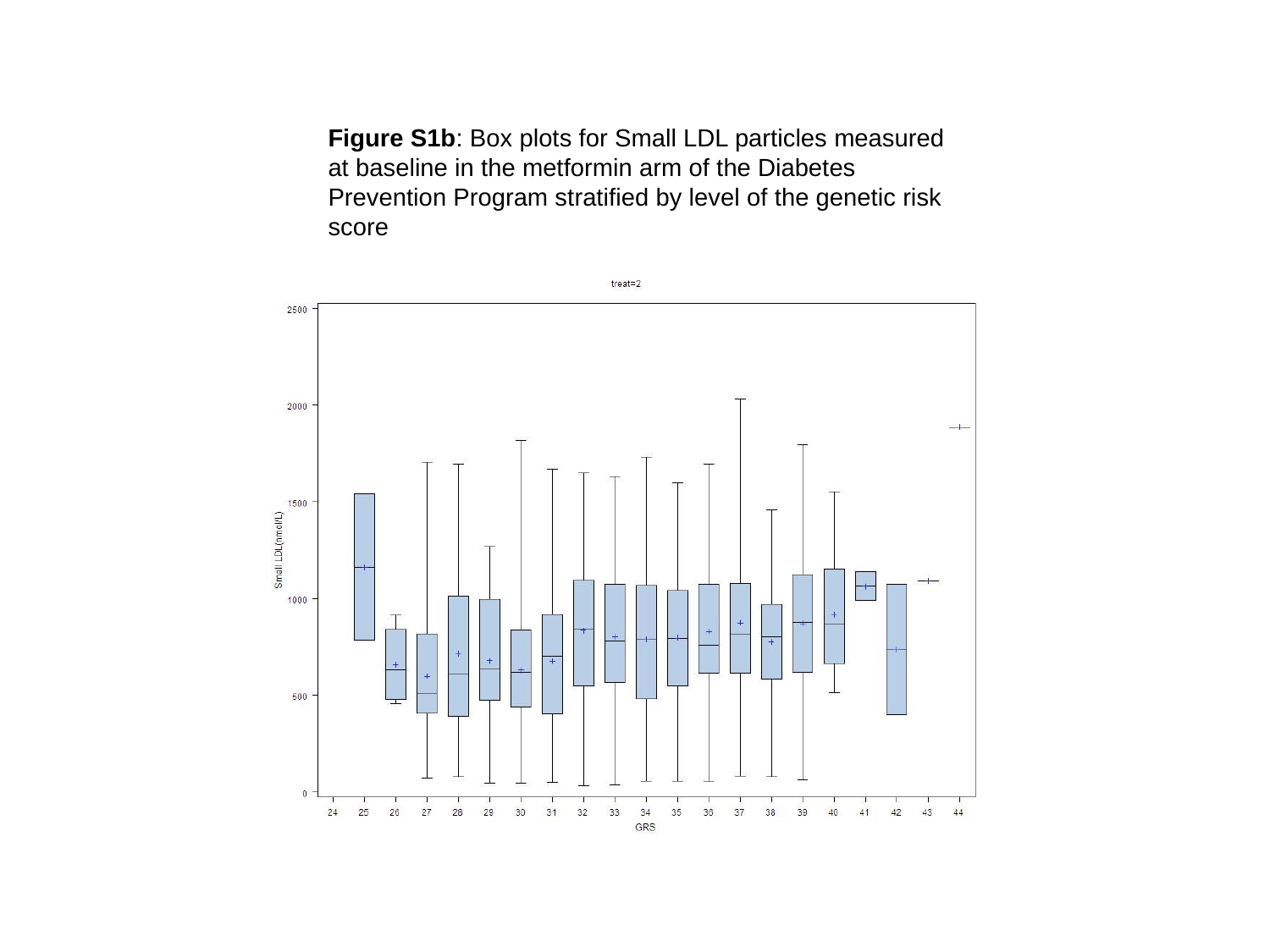

Figure S1b: Box plots for Small LDL particles measured at baseline in the metformin arm of the Diabetes Prevention Program stratified by level of the genetic risk score

## Slide 3
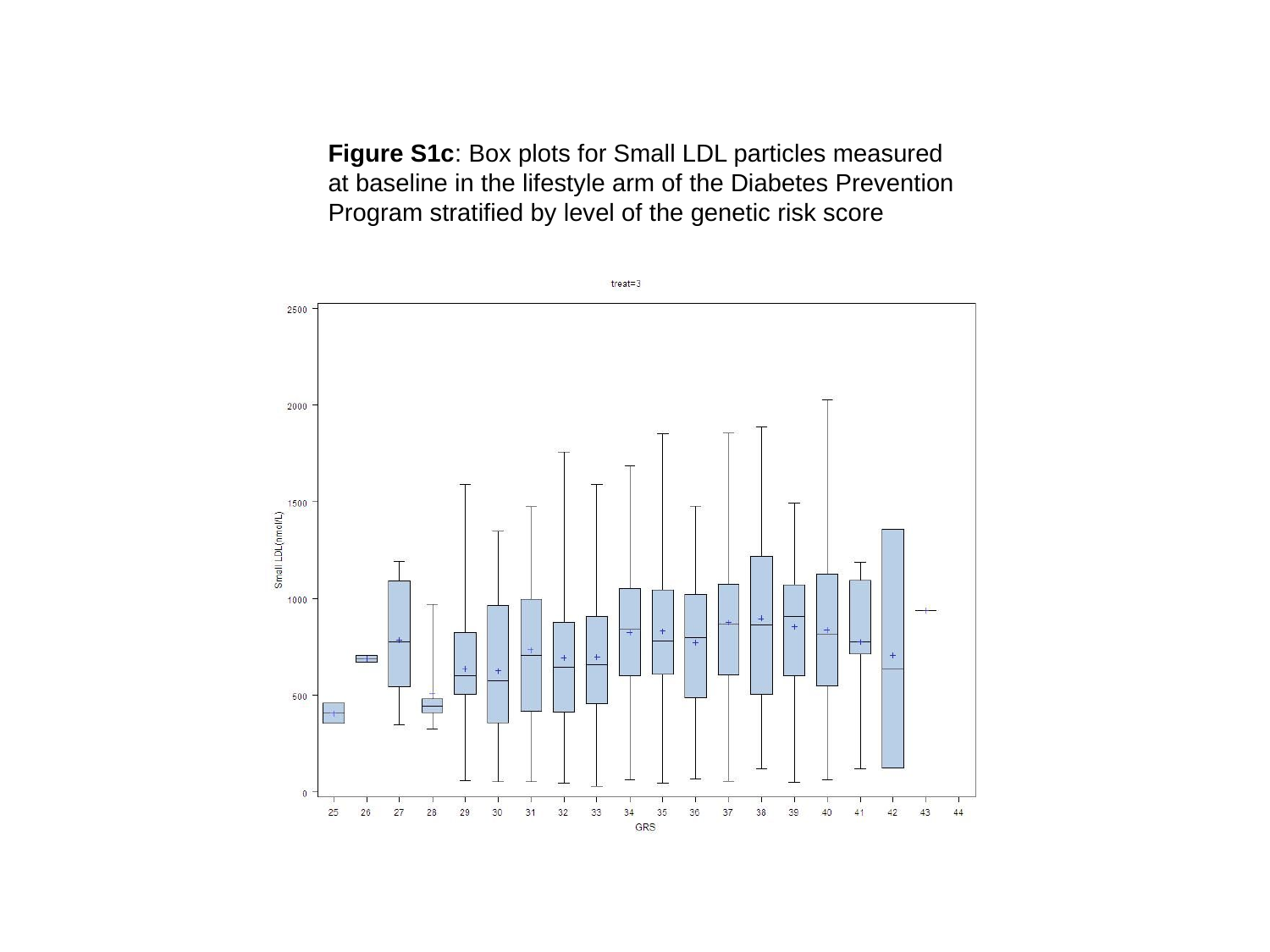

# Figure S1c: Box plots for Small LDL particles measured at baseline in the lifestyle arm of the Diabetes Prevention Program stratified by level of the genetic risk score

## Slide 4
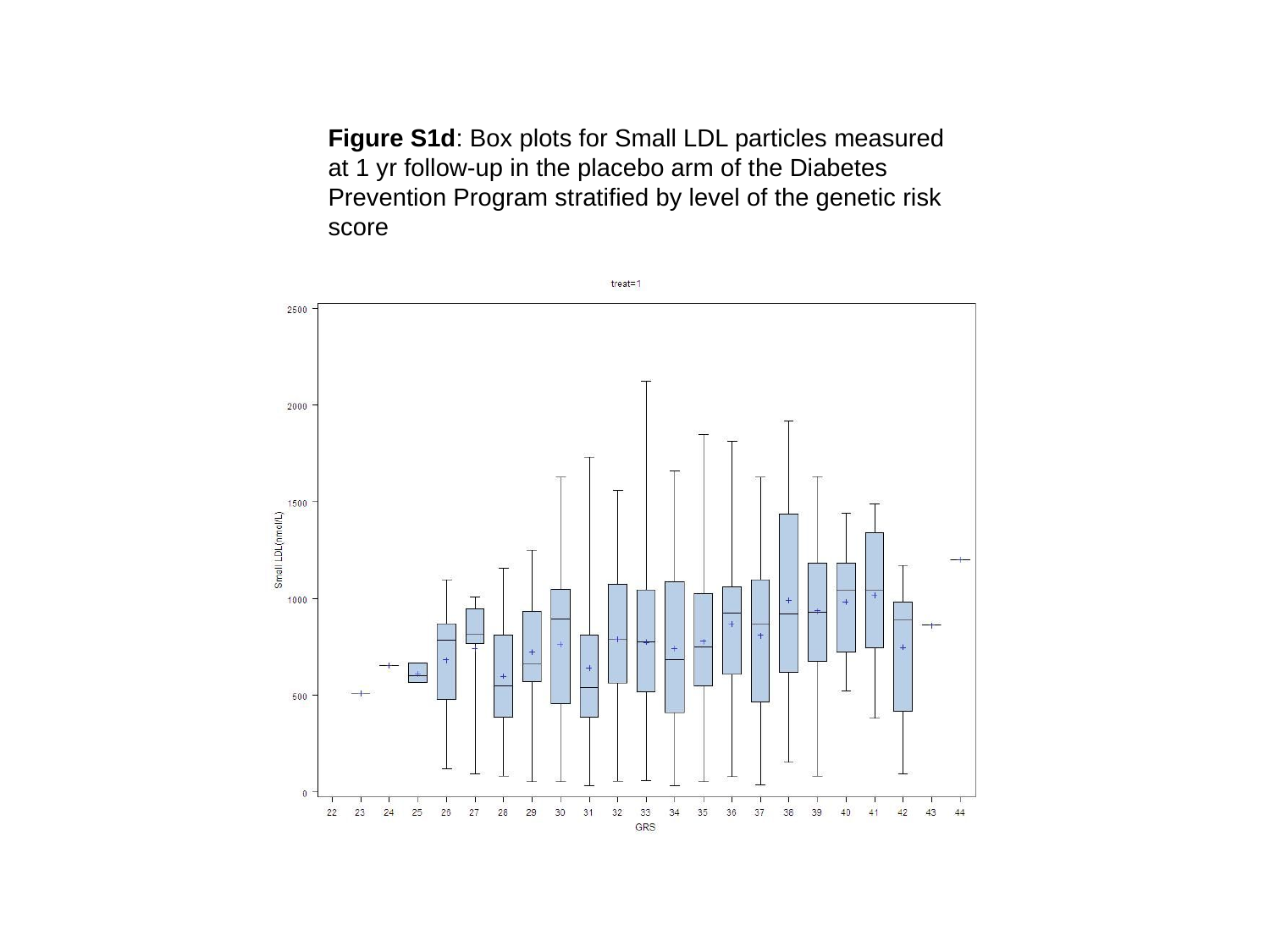

# Figure S1d: Box plots for Small LDL particles measured at 1 yr follow-up in the placebo arm of the Diabetes Prevention Program stratified by level of the genetic risk score

## Slide 5
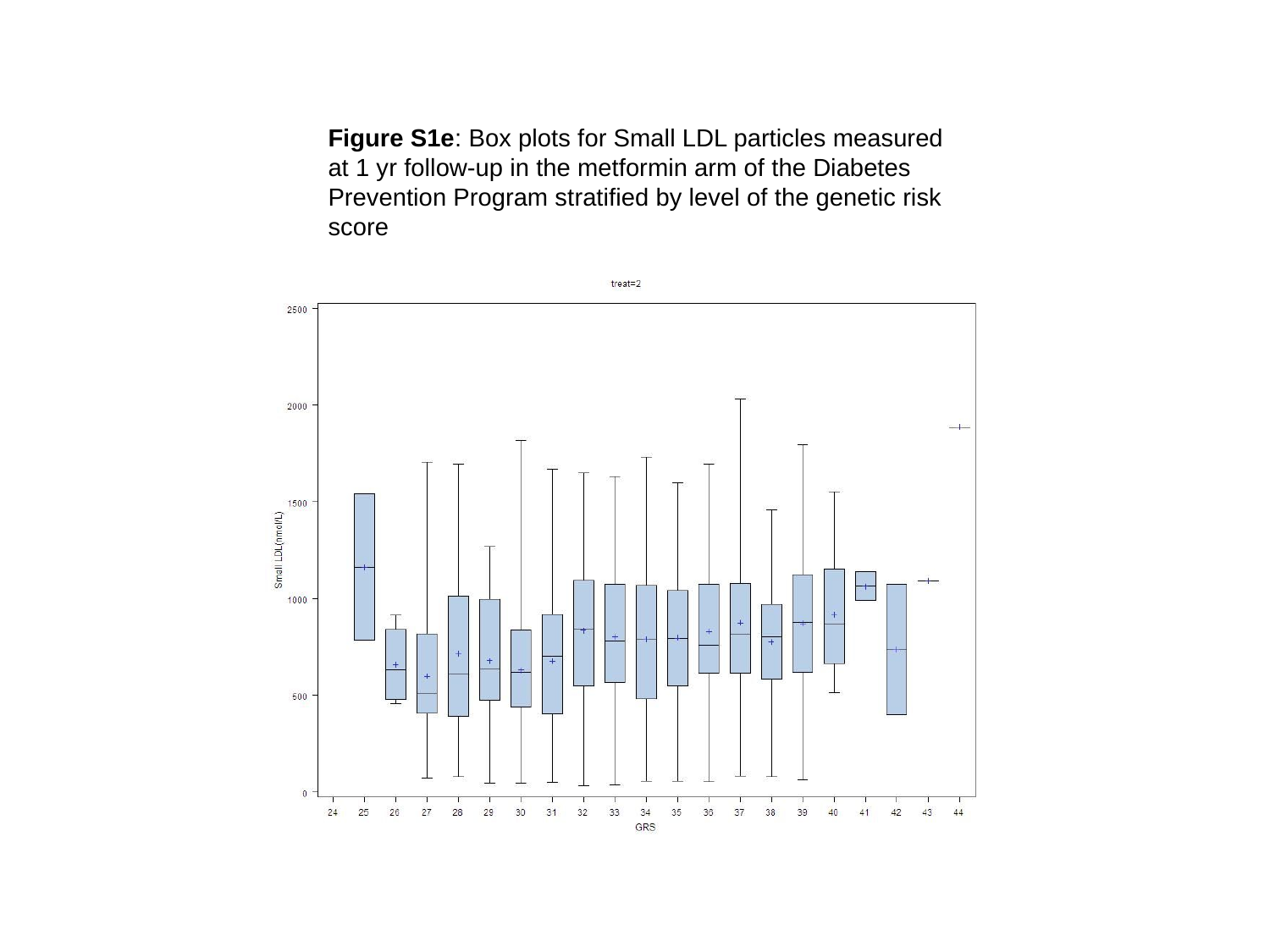

# Figure S1e: Box plots for Small LDL particles measured at 1 yr follow-up in the metformin arm of the Diabetes Prevention Program stratified by level of the genetic risk score

## Slide 6
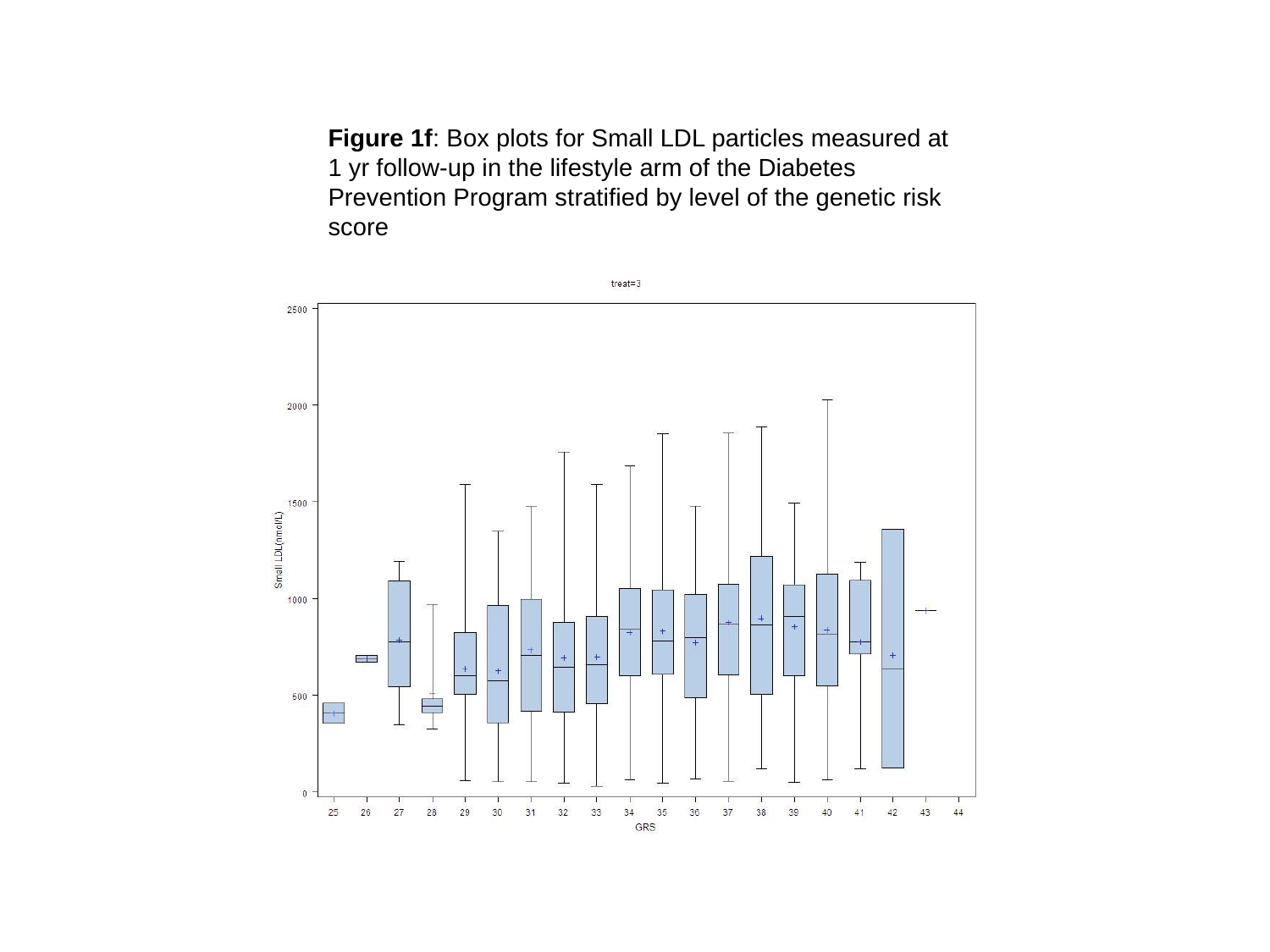

# Figure 1f: Box plots for Small LDL particles measured at 1 yr follow-up in the lifestyle arm of the Diabetes Prevention Program stratified by level of the genetic risk score
