# Supplementary material for: Genetic Modulation of Lipid Profiles following Lifestyle Modification or Metformin Treatment: The Diabetes Prevention Program
Source: PLoS Genet. 2012 Aug 30;8(8):e1002895. doi: 10.1371/journal.pgen.1002895 (PMC3431328; doi:10.1371/journal.pgen.1002895)
Supplement: Table S1 — ≤Details of individual SNP replication results (n2,843). The table compares results for each of the SNP loci in published meta-analysis and elsewhere with those reported here in the DPP. Analyses and means adjusted for age, sex, ethnicity and BMI. (DOCX) [file pgen.1002895.s002.docx]

## Table S1: Details of Single SNP Replication Results (n ≤ 2,843)

First identified in meta-analysis:

| Trait | Chrom | **SNP** | meta-analysis p-value | metaN | interval kb (genes) | *genes* | FHS alleles | FHS effect | DPP effect | DPP  General  p | DPP  Additive  p | Direction |
| --- | --- | --- | --- | --- | --- | --- | --- | --- | --- | --- | --- | --- |
| *LDL* | *2p21* | ***rs6544713*** | *2x10^-20^* | *23,456* | *52 (2)* | *ABCG8* | *C, T (0.32)^c^* | *0.15 (0.02)* | *2.31 (1.02)* | ***0.05*** | ***0.02*** | ***Same*** |
| LDL | 5q23 | **rs1501908** | 1x10^-11^ | 27,280 | 153 (2) | *TIMD4-HAVCR1* | C, G (0.37) | -0.07 (0.02 | -1.90 (0.92) | 0.12 | **0.04** | **Same** |
| LDL | 20q12 | **rs6102059** | 4x10^-9^ | 28,895 | 104 (0) | *MAFB* | C, T (0.32)^c^ | -0.06 (0.02) | -2.58 (0.94) | **0.01** | **0.006** | **Same** |
| LDL | 12q24 | **rs2650000** | 2x10^-8^ | 39,340 | 112 (3) | *TCF1* | C, A (0.36) | 0.07 (0.02) | 0.64 (0.97) | 0.77 | 0.51 | Same |
| HDL | 11q12 | **rs174547** | 2x10^-12^ | 40,330 | 84 (5) | *FADS1*-*FADS2*-*FADS3* | T, C (0.33) | -0.09 (0.02) | -0.010 (0.007) | 0.30 | 0.15 | Same |
| HDL | 16q22 | **rs2271293** | 9x10^-13^ | 31,946 | 620 (25) | *LCAT* | G, A (0.11) | 0.07 (0.03) | 0.020 (0.010) | **0.04** | **0.04** | **Same** |
| HDL | 9p22 | **rs471364** | 3x10^-10^ | 40,414 | 55 (1) | *TTC39B* | T, C (0.12) | -0.08 (0.03) | 0.001 (0.009) | 0.78 | 0.90 | Opposite |
| HDL | 20q13 | **rs1800961** | 8x10^-10^ | 30,714 | 24 (1) | *HNF4A* | C, T (0.03) | -0.19 (0.05) | -0.06 (0.02) | **0.004** | **0.002** | **Same** |
| HDL | 20q13 | **rs7679** | 4x10^-9^ | 40,248 | 141 (5) | *PLTP* | T, C (0.19) | -0.07 (0.02) | -0.016 (0.009) | 0.12 | 0.07 | Same |
| HDL | 19p13 | **rs2967605** | 1x10^-8^ | 35,151 | 155 (5) | *ANGPTL4* | C, T (0.16)^c^ | -0.12 (0.04) | -0.001 (0.008) | 0.82 | 0.88 | Same |
| TG | 11q12 | **rs174547** | 2x10^-14^ | 38,846 | 84 (5) | *FADS1*-*FADS2*-*FADS3* | T, C (0.33) | 0.06 (0.02) | 0.044 (0.015) | **0.002** | **0.004** | **Same** |
| TG | 20q13 | **rs7679** | 7x10^-11^ | 38,561 | 141 (5) | *PLTP* | T, C (0.19) | 0.07 (0.02) | 0.072 (0.019) | **0.0007** | **0.0002** | **Same** |
| TG | 8p23 | **rs7819412** | 3x10^-8^ | 33,336 | 550 (4) | *XKR6-AMAC1L2* | A, G (0.48) | -0.04 (0.02) | 0.026 (0.014) | 0.16 | 0.06 | Opposite |

Identified prior to meta-analysis:

| Trait | Chrom | **SNP** | meta-analysis p-value | metaN | interval kb (genes) | *genes* | FHS alleles | FHS effect | DPP effect | DPP  General  p | DPP  Additive  p | Direction |
| --- | --- | --- | --- | --- | --- | --- | --- | --- | --- | --- | --- | --- |
| LDL | 1p13 | **rs12740374** | 2x10^-42^ | 19,648 | 85 (4) | *CELSR2*, *PSRC1*, *SORT1* | G, T (0.21) | -0.23 (0.02) | -7.62 (1.04) | **4x10^-13^** | **3x10^-13^** | **Same** |
| LDL | 2p24 | **rs515135** | 5x10^-29^ | 19,648 | 214 (1) | *APOB* | C, T (0.20) | -0.16 (0.02) | -3.22 (1.04) | **0.001** | **0.002** | **Same** |
| LDL | 19q13 | **rs4420638** | 4x10^-27^ | 11,881 | 79 (4) | *APOE*-*C1*-*C4*-*C2* | A, G (0.16) | 0.29 (0.06) | 5.95 (1.19) | **4x10^-6^** | **7x10^-7^** | **Same** |
| LDL | 19p13 | **rs6511720** | 2x10^-26^ | 19,648 | 30 (1) | *LDLR* | G, T (0.10) | -0.26 (0.04) | -5.43 (1.38) | **0.0003** | **9x10^-5^** | **Same** |
| LDL | 5q13 | **rs3846663** | 8x10^-12^ | 19,648 | 476 (4) | *HMGCR* | C, T (0.38) | 0.07 (0.02) | 3.30 (0.91) | **0.002** | **0.0003** | **Same** |
| LDL | 19p13 | **rs10401969** | 2x10^-8^ | 19,648 | 503 (18) | *CSPG3, CILP2, PBX4* | T, C (0.06) | -0.05 (0.04) | -0.11 (1.56) | 1.00 | 0.94 | Same |
| LDL | 1p32 | **rs11206510** | 4x10^-8^ | 19,629 | 16 (1) | *PCSK9* | T, C (0.19) | -0.09 (0.02) | -0.048 (1.19) | 0.97 | 0.97 | Same |
| HDL | 16q13 | **rs173539** | 4x10^-75^ | 19,794 | 36 (1) | *CETP* | C, T (0.32) | 0.25 (0.02) | -- | -- | -- | -- |
| HDL | 16q13 | **rs247616** | proxy for rs173539 |  |  | *CETP* | C,T |  | 0.06  (0.01) | **5x10^-17^** | **1x10^-17^** | **Same** |
| HDL | 8p21 | **rs12678919** | 2x10^-34^ | 19,794 | 126 (1) | *LPL* | A, G (0.10) | 0.23 (0.03) | 0.05 (0.01) | **10x10^-7^** | **5x10^-7^** | **Same** |
| HDL | 15q22 | **rs10468017** | 8x10^-23^ | 19,794 | 47 (0) | *LIPC* | C, T (0.30) | 0.10 (0.02) | 0.03 (0.01) | **0.001** | **0.0003** | **Same** |
| HDL | 18q21 | **rs4939883** | 7x10^-15^ | 19,785 | 128 (1) | *LIPG* | C, T (0.17) | -0.14 (0.02) | -0.03 (0.01) | **0.001** | **0.0003** | **Same** |
| HDL | 11q23 | **rs964184** | 1x10^-12^ | 19,794 | 138 (3) | *APOA1-C3-A4-A5* | C, G (0.14) | -0.17 (0.03) | -0.03 (0.01) | **5x10^-5^** | **1x10^-5^** | **Same** |
| HDL | 12q24 | **rs2338104** | 1x10^-10^ | 19,793 | 315 (5) | *MMAB*, *MVK* | G, C (0.45) | -0.07 (0.02) | -0.01 (0.01) | 0.19 | 0.08 | Same |
| HDL | 9q31 | **rs1883025** | 1x10^-9^ | 19,371 | 48 (1) | *ABCA1* | C, T (0.26) | -0.08 (0.02) | -0.03 (0.01) | **0.0007** | **0.0001** | **Same** |
| HDL | 1q42 | **rs4846914** | 4x10^-8^ | 19,794 | 53 (1) | *GALNT2* | A, G (0.40) | -0.05 (0.02) | -0.02 (0.01) | **0.02** | **0.004** | **Same** |
| TG | 11q23 | **rs964184** | 4x10^-62^ | 19,840 | 138 (3) | *APOA1-C3-A4-A5* | C, G (0.14) | 0.30 (0.03) | 0.138 (0.017) | **4x10^-15^** | **4x10^-16^** | **Same** |
| TG | 8p21 | **rs12678919** | 2x10^-41^ | 19,840 | 126 (1) | *LPL* | A, G (0.10) | -0.25 (0.03) | -0.101 (0.022) | **3x10^-5^** | **5x10^-6^** | **Same** |
| TG | 2p23 | **rs1260326** | 2x10^-31^ | 19,840 | 465 (22) | *GCKR* | C, T (0.45) | 0.12 (0.02) | 0.085 (0.014) | **2x10^-8^** | **3x10^-9^** | **Same** |
| TG | 8q24 | **rs2954029** | 3x10^-19^ | 19,840 | 35 (0) | *TRIB1* | A, T (0.44) | -0.11 (0.02) | -0.042 (0.013) | **0.007** | **0.002** | **Same** |
| TG | 7q11 | **rs714052** | 3x10^-15^ | 19,840 | 254 (5) | *MLXIPL* | A, G (0.12) | -0.16 (0.03) | -0.034 (0.023) | 0.26 | 0.14 | Same |
| TG | 2p24 | **rs7557067** | 9x10^-12^ | 19,840 | 149 (1) | *APOB* | A, G (0.22) | -0.08  (0.02) | -0.022 (0.016) | 0.35 | 0.16 | Opposite |
| TG | 19p13 | **rs17216525** | 4x10^-11^ | 19,840 | 448 (15) | *CSPG3*, *CILP2*, *PBX4* | C, T (0.07) | -0.11 (0.03) | -0.102 (0.028 | **0.0004** | **0.0002** | **Same** |
| TG | 1p31 | **rs10889353** | 3x10^-7^ | 19,834 | 305 (3) | *ANGPTL3* | A, C (0.33) | -0.05 (0.02) | -0.046 (0.014) | **0.002** | **0.001** | **Same** |

FHS: Framingham Heart Study results; Effects indicated are for second/original minor allele
