# Supplementary material for: Genetic Modulation of Lipid Profiles following Lifestyle Modification or Metformin Treatment: The Diabetes Prevention Program
Source: PLoS Genet. 2012 Aug 30;8(8):e1002895. doi: 10.1371/journal.pgen.1002895 (PMC3431328; doi:10.1371/journal.pgen.1002895)
Supplement: Table S2 — <Associations of individual SNPs with lipid or lipoprotein traits significant at the Bonferroni-significant p-value of 0.0001 for additive model (see Table 1 for units). Shown are geometric means for all traits except LDL-C, for which arithmetic means are shown. All analyses and means adjusted for age, sex, ethnicity and BMI. (DOCX) [file pgen.1002895.s003.docx]

## Table S2: Associations of single SNPs with lipid or lipoprotein traits significant at the Bonferroni-significant p-value of <0.0001 for additive model (see Table 1 for units). Shown are geometric means for all traits except LDL-C, for which arithmetic means are shown. All analyses and means adjusted for age, sex, ethnicity and BMI.

| Loc | SNP **Gene(s)** | Major/minorallele (1/2) | N | MAF | Trait | 1/1 **Mean (95% CI)** | | 1/2Mean (95% CI) | 2/2 **Mean (95% CI)** | Generalp-value | Additivep-value |
| --- | --- | --- | --- | --- | --- | --- | --- | --- | --- | --- | --- |
| 1p13 | rs12740374 | G/T | 2,757 | 0.22 | Cholesterol | 204 (202 – 205) | | 194 (192 – 196) | 192 (187 – 198) | 2×10^-11^ | 2×10^-11^ |
|  | *CELSR2, PSRC1, SORT1* |  |  |  | LDL-C | 129 (127 – 130) | | 119 (117 – 121) | 117 (112 – 122) | 5×10^-13^ | 3×10^-13^ |
|  |  |  |  |  | LDL Particles | 1354 (1331 – 1377) | | 1278 (1249 – 1307) | 1246 (1176 – 1320) | 4×10^-5^ | 9×10^-6^ |
| 2p23 | rs1260326 | C/T | 2,818 | 0.34 | TG | 133 (130 – 137) | | 145 (141 – 149) | 159 (150 – 167) | 2×10^-8^ | 3×10^-9^ |
|  | *GCKR* |  |  |  | LDL Size | 0.265 (0.264 – 0.267) | | 0.262 (0.260 – 0.264) | 0.258 (0.255 – 0.262) | 0.0004 | 8×10^-5^ |
|  |  |  |  |  | VLDL Particles | 56 (54 – 58) | | 61 (59 – 63) | 64 (60 – 69) | 0.0002 | 4×10^-5^ |
|  |  |  |  |  | Large VLDL | 4.48 (4.20 – 4.78) | | 5.62 (5.26 – 6.01) | 6.79 (6.01 – 7.67) | 1×10^-9^ | 2×10^-10^ |
|  |  |  |  |  | VLDL Size | 51.81 (52.27 – 52.35) | | 53.25 (52.69 – 53.81) | 54.88 (53.83 – 55.95) | 6 x 10^-7^ | 9 x 10^-8^ |
| 2p24 | rs7557067 *APOB* | A/G | 2,756 | 0.25 | VLDL Particles | 62 (60 – 63) | | 57 (55 – 59) | 53 (48 – 58) | 0.0003 | 8×10^-5^ |
| 8p21 | rs12678919 | A/G | 2,753 | 0.10 | HDL-C | 43.7 (43.3 – 44.1) | | 45.6 (44.7 – 46.5) | 50.9 (47.2 – 55.0) | 1×10^-6^ | 5×10^-7^ |
|  | *LPL* |  |  |  | TG | 144 (141 – 147) | | 130 (125 – 136) | 117 (99 – 139) | 3×10^-5^ | 5×10^-6^ |
|  |  |  |  |  | LDL Size | 0.262 (0.261 – 0.263) | | 0.267 (0.265 – 0.270) | 0.273 (0.263 – 0.283) | 0.0002 | 4×10^-5^ |
|  |  |  |  |  | VLDL Particles | 60 (59 – 62) | | 54 (51 – 57) | 49 (40 – 60) | 0.0003 | 6 × 10^-5^ |
| 11q23 | rs964184 | C/G | 2,753 | 0.19 | HDL-C | 44.6 (44.2 – 45.1) | | 43.4 (42.8 – 44.1) | 41.1 (39.5 – 42.8) | 5×10^-5^ | 1×10^-5^ |
|  | *APOA1-C3-A4-A5* |  |  |  | TG | 134 (131 – 137) | | 153 (148 – 159) | 176 (162 – 193) | 4×10^-15^ | 4×10^-16^ |
|  |  |  |  |  | LDL Size | 0.266 (0.265 – 0.267) | | 0.258 (0.256 – 0.260) | 0.249 (0.244 – 0.254) | 3×10^-16^ | 3×10^-17^ |
|  |  |  |  |  | VLDL Particles | 57 (55 – 58) | | 63 (61 – 66) | 72 (65 – 80) | 3×10^-7^ | 4×10^-8^ |
|  |  |  |  |  | Large VLDL | 4.79 (4.55 – 5.05) | | 5.90 (5.45 – 6.39) | 7.28 (6.08 – 8.94) | 2×10^-7^ | 3×10^-8^ |
| 15q22 | rs10468017 | C/T | 2,734 | 0.24 | Large HDL | 3.16 (3.06 – 3.28) | | 3.49 (3.34 – 3.65) | 3.96 (3.58 – 4.38) | 8×10^-6^ | 1×10^-6^ |
|  | *LIPC* |  |  |  | Small HDL | 18.91 (18.60 – 19.22) | | 18.19 (17.82 – 18.57) | 16.78 (16.01 – 17.59) | 3×10^-6^ | 1×10^-6^ |
|  |  |  |  |  | HDL Size | 8.83 (8.80 – 8.85) | | 8.89 (8.86 – 8.92) | 9.00 (8.93 – 9.06) | 6×10^-7^ | 1×10^-7^ |
| 16q13 | rs247616 | C/T | 2,799 | 0.30 | HDL-C | | 42.7 (42.3 – 43.3) | 44.8 (44.2 – 45.4) | 48.4 (47.1 – 49.7) | 5×10^-17^ | 1×10^-17^ |
|  | *CETP* |  |  |  | LDL Size | | 0.260 (0.259 – 0.262) | 0.265 (0.263 – 0.266) | 0.270 (0.267 – 0.274) | 1×10^-7^ | 2×10^-8^ |
|  |  |  |  |  | Large HDL | | 3.20 (3.08 – 3.32) | 3.32 (3.18 – 3.45) | 4.05 (3.72 – 4.41) | 4×10^-6^ | 2×10^-5^ |
| 19p13 | rs10401969 | T/C | 2,756 | 0.09 | Large VLDL | | 5.43 (5.18 – 5.69) | 4.26 (3.83 – 4.74) | 4.07 (2.61 – 6.35) | 0.0002 | 4×10^-5^ |
|  | *CSPG3, CILP2, PBX4* |  |  |  | VLDL Size | | 53.18 (52.78 – 53.58) | 50.94 (50.07 – 51.82) | 49.55 (46.20 – 53.14) | 1 x 10^-5^ | 2×10^-6^ |
| 19p13 | rs17216525 | C/T | 2,759 | 0.06 | Large VLDL | | 5.38 (5.14 – 5.63) | 4.15 (3.67 – 4.70) | 3.41 (1.62 – 7.17) | 0.0004 | 7×10^-5^ |
|  | *CSPG3, CILP2, PBX4* |  |  |  | VLDL Size | | 53.06 (52.68 – 53.45) | 50.86 (49.86 – 51.88) | 48.48 (43.13 – 54.50) | 0.0002 | 3×10^-5^ |
| 19p13 | rs6511720 *LDLR* | G/T | 2,727 | 0.12 | LDL-C | | 126 (125 – 128) | 121 (119 – 124) | 112 (101 – 123) | 0.0003 | 9×10^-5^ |
| 19q13 | rs4420638 | A/G | 2,753 | 0.16 | Cholesterol | | 198 (196 – 199) | 205 (202 – 207) | 211 (203 – 220) | 1×10^-6^ | 2×10^-7^ |
|  | *APOE-C1-C4-C2* |  |  |  | LDL-C | | 123 (122 – 125) | 129 (127 – 131) | 135 (128 – 143) | 4×10^-6^ | 7×10^-7^ |
|  |  |  |  |  | LDL Size | | 0.264 (0.263 – 0.266) | 0.260 (0.258 – 0.262) | 0.254 (0.248 – 0.261) | 7×10^-5^ | 1×10^-5^ |
|  |  |  |  |  | LDL Particles | | 1295 (1275 – 1315) | 1377 (1342 – 1413) | 1527 (1408 – 1656) | 9×10^-7^ | 2×10^-7^ |
| 20q13 | rs7679 | T/C | 2,757 | 0.14 | Small HDL | | 17.93 (17.68 – 18.18) | 20.14 (19.62 – 20.68) | 21.89 (20.35 – 23.54) | 4×10^-17^ | 4×10^-18^ |
|  | *PLTP* |  |  |  | HDL Particles | | 34.07 (33.78 – 34.37) | 35.10 (34.54 – 35.66) | 36.78 (35.18 – 38.46) | 9×10^-5^ | 2×10^-5^ |
