# Supplementary material for: Genetic Modulation of Lipid Profiles following Lifestyle Modification or Metformin Treatment: The Diabetes Prevention Program
Source: PLoS Genet. 2012 Aug 30;8(8):e1002895. doi: 10.1371/journal.pgen.1002895 (PMC3431328; doi:10.1371/journal.pgen.1002895)
Supplement: Table S3 — = Associations of individual SNPs with lipid or lipoprotein traits measured at baseline in White participants from the DPP (n1,565). Analyses were performed to determine whether population stratification owing to the mutliethnic nature of the DPP is likely to confound the associations reported in the main analyses. The comparability of the results in White DPP participants with the main analyses indicates that confoudning by population stratification is unlikely to underly the main results reported here. Analyses and means adjusted for age, sex, and BMI. (DOCX) [file pgen.1002895.s004.docx]

**Table S3:** Associations of individual SNPs with lipid or lipoprotein traits measured at baseline in White participants from the DPP (n = 1,565). Analyses were performed to determine whether population stratification owing to the mutliethnic nature of the DPP is likely to confound the associations reported in the main analyses. The comparability of the results in White DPP participants with the main analyses indicates that confoudning by population stratification is unlikely to underly the main results reported here. Analyses and means adjusted for age, sex, and BMI.

| Trait | Q1 | Q2 | Q3 | Q4 | %diff** | Partial r* | Beta±SE/ unit | p-value*** |
| --- | --- | --- | --- | --- | --- | --- | --- | --- |
|  | (22 - 32) | (33 - 34) | (35 - 36) | (37 - 44) |  |  |  |  |
| n | 505 | 386 | 351 | 323 | -- | -- | -- | -- |
| Chol (mg/dl) | 198 | 202 | 203 | 208 | 5% | 0.10 | +0.0062 ± 0.0014 | **5 x 10^-6^** |
|  | (195-201) | (199-205) | (199-206) | (204-212) |  |  |  |  |
| LDL-C (mg/dl) | 121 | 125 | 125 | 129 | 7% | 0.10 | +0.95 ± 0.25 | **1 x 10^-4^** |
|  | (118-124) | (122-129) | (122-129) | (126-132) |  |  |  |  |
| HDL-C (mg/dl) | 47 | 45 | 45 | 43 | 9% | -0.18 | -0.0122 ± 0.0018 | **2 x 10^-11^** |
|  | (47-48) | (44-46) | (43-46) | (42-44) |  |  |  |  |
| TG (mg/dl) | 140 | 153 | 160 | 173 | 24% | 0.16 | +0.0264 ± 0.0040 | **4 x 10^-11^** |
|  | (134-147) | (145-160) | (152-168) | (164-183) |  |  |  |  |
| LDL size (nm) | 0.266 | 0.262 | 0.258 | 0.254 | 5% | -0.16 | -0.0060 ± 0.0009 | **3 x 10^-11^** |
|  | (0.263-0.268) | (0.259-0.265) | (0.255-0.261) | (0.251-0.257) |  |  |  |  |
| Total VLDL particles (nmol/L) | 58 | 67 | 68 | 73 | 26% | 0.19 | +0.0292 ± 0.0047 | **5 x 10^-10^** |
|  | (55-61) | (63-71) | (64-72) | (69-78) |  |  |  |  |
| Large VLDL particles (nmol/L) | 5.21 | 5.98 | 7.06 | 7.86 | 51% | 0.16 | +0.0507 ± 0.0092 | **4 x 10^-8^** |
|  | (4.71-5.75) | (5.34-6.7) | (6.26-7.97) | (6.92-8.92) |  |  |  |  |
| Total LDL particles (nmol/L) | 1297 | 1371 | 1373 | 1454 | 12% | 0.15 | +0.0135 ± 0.0026 | **3 x 10^-7^** |
|  | (1260-1335) | (1327-1416) | (1327-1422) | (1402-1508) |  |  |  |  |
| Small LDL particles (nmol/L) | 574 | 709 | 754 | 811 | 41% | 0.18 | +0.0420 ± 0.0068 | **1 x 10^-9^** |
|  | (532-618) | (652-772) | (690-825) | (738-891) |  |  |  |  |
| Large HDL particles (µmol/L) | 3.51 | 3.06 | 2.99 | 2.81 | 20% | -0.16 | -0.0263 ± 0.0055 | **2 x 10^-6^** |
|  | (3.31-3.73) | (2.86-3.28) | (2.78-3.21) | (2.61-3.04) |  |  |  |  |
| Small HDL particles (µmol/L) | 18.39 | 18.71 | 19.28 | 19.57 | 6% | 0.11 | +0.0090 ± 0.0025 | **0.0004** |
|  | (17.89-18.91) | (18.13-19.32) | (18.65-19.94) | (18.89-20.27) |  |  |  |  |
| HDL size (nm) | 8.87 | 8.8 | 8.79 | 8.78 | 1% | -0.10 | -0.0012 ± 0.0004 | **0.001** |
|  | (8.83-8.91) | (8.76-8.84) | (8.75-8.83) | (8.73-8.83) |  |  |  |  |
| VLDL size (nm) | 52.7 | 53.03 | 54.56 | 55.04 | 4% | 0.11 | +0.0052 ± 0.0015 | **5 x 10^-4^** |
|  | (51.84-53.57) | (52.06-54.02) | (53.5-55.65) | (53.91-56.18) |  |  |  |  |
| Total HDL particles (µmol/L) | 35.3 | 34.64 | 35.07 | 34.3 | 3% | -0.05 | -0.0024 ± 0.0016 | 0.12 |
|  | (34.7-35.91) | (33.98-35.32) | (34.35-35.79) | (33.57-35.06) |  |  |  |  |

* Quartiles were assigned separately in each ethnic group, leading to slight overlap in quartile ranges of number of risk alleles. Traits are age-, sex-, and BMI-adjusted geometric means and 95% confidence intervals, except LDL-C, for which arithmetic mean and 95% confidence interval are shown.

** Percent difference between Q4 and Q1 in reference to Q1

*** Partial r and p-value based on analysis of GRS as a quantitative covariate with adjustment for age, sex, and BMI.
